# A Phylogenetic Analysis of the Globins in Fungi

**DOI:** 10.1371/journal.pone.0031856

**Published:** 2012-02-27

**Authors:** David Hoogewijs, Sylvia Dewilde, Andy Vierstraete, Luc Moens, Serge N. Vinogradov

**Affiliations:** 1 Institute of Physiology and Zürich Center for Integrative Human Physiology (ZIHP), University of Zürich, Zürich, Switzerland; 2 Department of Biomedical Sciences, University of Antwerp, Antwerp, Belgium; 3 Department of Biology, Ghent University, Ghent, Belgium; 4 Department of Biochemistry and Molecular Biology, Wayne State University School of Medicine, Detroit, Michigan, United States of America; Nanjing Agricultural University, China

## Abstract

**Background:**

All globins belong to one of three families: the F (flavohemoglobin) and S (sensor) families that exhibit the canonical 3/3 α-helical fold, and the T (truncated 3/3 fold) globins characterized by a shortened 2/2 α-helical fold. All eukaryote 3/3 hemoglobins are related to the bacterial single domain F globins. It is known that Fungi contain flavohemoglobins and single domain S globins. Our aims are to provide a census of fungal globins and to examine their relationships to bacterial globins.

**Results:**

Examination of 165 genomes revealed that globins are present in >90% of Ascomycota and ∼60% of Basidiomycota genomes. The S globins occur in Blastocladiomycota and Chytridiomycota in addition to the phyla that have FHbs. Unexpectedly, group 1 T globins were found in one Blastocladiomycota and one Chytridiomycota genome. Phylogenetic analyses were carried out on the fungal globins, alone and aligned with representative bacterial globins. The Saccharomycetes and Sordariomycetes with two FHbs form two widely divergent clusters separated by the remaining fungal sequences. One of the Saccharomycete groups represents a new subfamily of FHbs, comprising a previously unknown N-terminal and a FHb missing the C-terminal moiety of its reductase domain. The two Saccharomycete groups also form two clusters in the presence of bacterial FHbs; the surrounding bacterial sequences are dominated by Proteobacteria and Bacilli (Firmicutes). The remaining fungal FHbs cluster with Proteobacteria and Actinobacteria. The Sgbs cluster separately from their bacterial counterparts, except for the intercalation of two Planctomycetes and a Proteobacterium between the Fungi incertae sedis and the Blastocladiomycota and Chytridiomycota.

**Conclusion:**

Our results are compatible with a model of globin evolution put forward earlier, which proposed that eukaryote F, S and T globins originated via horizontal gene transfer of their bacterial counterparts to the eukaryote ancestor, resulting from the endosymbiotic events responsible for the origin of mitochondria and chloroplasts.

## Introduction

The presence of hemeproteins capable of reversible reaction with oxygen in the yeasts *Saccharomyces* an*d Candida* and in the mold *Neurospora* was first reported by Keilin [1], [2]. Chance et al. showed the yeast hemeprotein to have a mass of about 50 kDa and to contain one heme and one flavin group [3]. Subsequently, the amino acid sequences of the flavohemoglobins (FHbs) from *Candida norvegensis* [4] and *Saccharomyces cerevisiae* [5] were determined, and found to be very similar to the *E. coli* HMP protein, concurrently discovered by R. Poole's group [6]. The accumulation of genomic information over the next decade and a half led to a substantial revision of globin distribution and phylogeny. It became clear that all bacterial globins could be classified into three families [7], [8]. Two of them, the F (flavohemoglobin) [9] and S (sensor) [10] families, share the familiar 3/3 α-helical myoglobin-fold characteristic of metazoan hemoglobins (Hbs). The third family encompasses the T (truncated Mb-fold) Hbs exhibiting an abbreviated 2/2 α-helical Mb-fold characterized by vestigial helices A and E; the T sequences are further subdivided into three groups (groups 1–3) [11], [12], [13]. All three globin families comprise chimeric and single domain globins. Furthermore, although the details of the phylogenetic relationships of the three bacterial globin families to each other and the many eukaryotic globin subfamilies remain to be elucidated [14], [15], it is reasonably evident that all eukaryote 3/3 Mb-fold globins, including vertebrate, plant and other metazoan Hbs are related to the bacterial F globins.

In this communication, we report on the known and novel putative globins identified in 165 fungal genomes and the results of molecular phylogenetic analyses of the fungal Hbs and their relationship to the bacterial and other eukaryote globins.

## Results

### Distribution of globins

**Figure 1:**
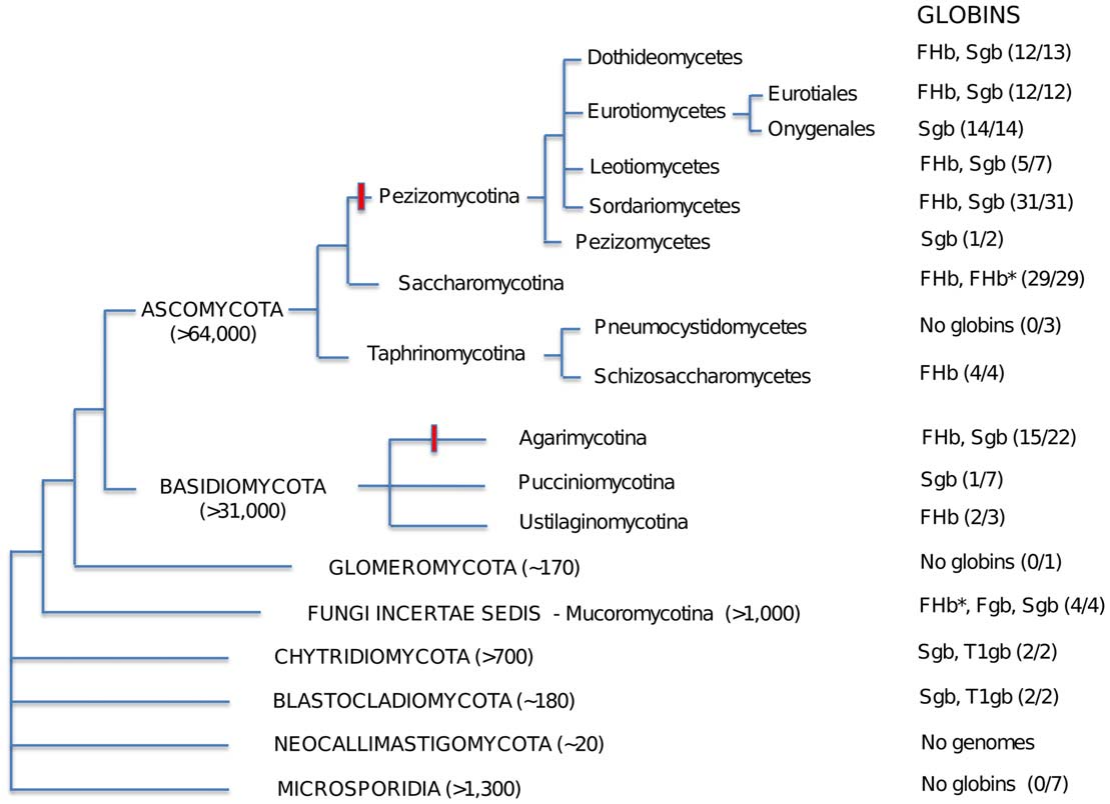
Diagrammatic representation of fungal phylogeny based on ref. 18 and of their putative globins. Estimates of the numbers of species in parentheses are from the *Dictionary of the Fungi* [23]. Red bar indicates multicellularity with differentiated tissues; FHb* - unknown N-terminal domain linked to a FHb with an incomplete reductase domain, T1gb - T globin group1. Ratios refer to the number of genomes containing globins versus the total number of genomes analyzed.

The distribution of globins in 165 fungal genomes is summarized in the diagrammatic representation of fungal phylogeny shown in Fig. 1. The names of the species and the globins identified are listed in Supplemental Data Table S1, employing NCBI Taxonomy (www.ncbi.nih.gov/Taxonomy/). The Ascomycota with 117 genomes are the most numerous and also exhibit a high percentage of genomes with globins (>90%), except for the Pneumocystidiomycetes, where 3 genomes are devoid of globins. The 33 Basidiomycota genomes have a much lower percentage of genomes with globins (∼59%). Noteworthy is the total absence of globins in Microsporidia and the single Glomeromycota genome. No genomic information is available for the Neocallimastigomycota. It should be pointed out that because about half of the analyzed genomes are not completed, it is not always clear if a given genome lacks globins.

Overall, the fungi have one or two types of globins homologous to members of the bacterial globin F and S families (Fig. 1 and Table S1). Except for the Muromycotina the F family is mostly represented by the complete chimeric FHbs, The fungal members of the S globin family are limited to single domain sensor globins (Sgbs). In Table S1, we differentiate between Sgbs, which are generally <260 residues and other larger chimeric proteins where in addition to the sensor domain (SD) there exists a C-terminal extension of about 100 residues or more, large enough to represent an unidentified domain. Among the Ascomycota, FHbs and Sgbs coexist except in the Onygenales (Eurotiomycetes) and Pezizomycetes that have only Sgbs and the Schizosaccharomycetes that have only FHbs. Among the Basidiomycota they coexist in the Agarimycotina, with an Sgb in the Pucciniamycotine, and only FHbs in the Ustilagomycotina.

An unexpected finding was the presence of two putative globins belonging to the bacterial T family group 1, one as a C-terminal domain in a large 1129 residue protein in *Allomyces macrogynus* (Blastocladiomycota) and one as a single domain globin in *Batrachochytrium dendrobatidis* (Chytridiomycota).

### Subfamilies of F globins

**Figure 2:**
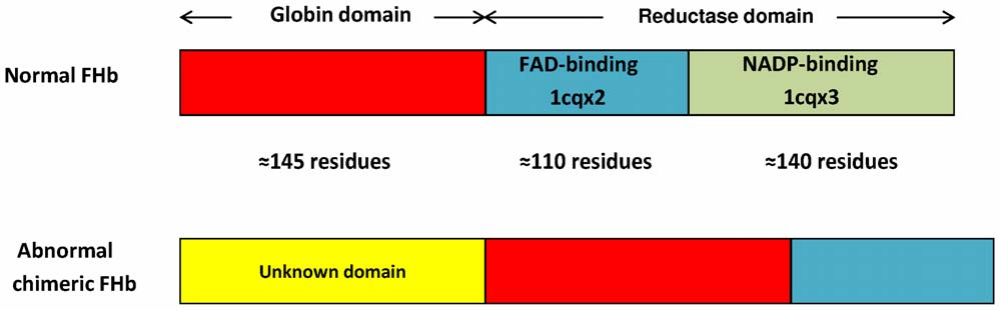
Diagrammatic representation of FHb structures. Schematically displayed structures are those of a normal FHb and of an abnormal chimeric FHb missing the C-terminal moiety of the reductase domain found in 21 of 29 Saccharomycotina genomes. The normal FHb is represented by *E. coli* FHb (PDB: 1gvh).

It is possible to distinguish four subfamilies of F globins in fungi. One, comprises single domain Fgbs present only in the Mucoromycotina (Fungi incertae sedis) (Table S1). All three genomes have 2 F globins: five of them have N- and C-terminal extensions. In contrast to the latter, the N-terminal extensions exceed 100 residues in three cases, and may represent unidentified domains. Apart from the normal 380 to 420 residue FHbs, 2 other subfamilies can be distinguished on the basis of N- and C-terminal extensions and defects in the reductase FHb domain. One subfamily is represented by a subset of FHbs within 21 of the 29 Saccharomycotina (Ascomycota) genomes that possess one to three chimeric proteins with an unidentified N-terminal and an incomplete FHb, missing the C-terminal portion of the reductase moiety (identified by an asterisk in Fig. 1). Fig. 2 illustrates diagrammatically the structures of a normal FHb and an abnormal chimeric FHb found in the majority of the Saccharomycete genomes. The structure of a typical FHb, the *E. coli* FHb (396aa; PDB: 1gvh) has three domains, with the ferredoxin reductase-like domain comprising domains cqx2 and cqx3, representing the FAD-binding and NADP-binding domains, respectively. FUGUE searches identify FHbs by providing Z scores >6 for the globin, the cqx2 and the cqx3 domains. The absence of cqx3 is diagnostic of the incomplete FHbs.

Another subfamily represents FHbs that have >25 residue N-terminal extensions, of which the first ∼20 residues can be identified as a possible signal peptide via MITOprot and SignalP (www.expasy.org/tools) (Table S1). These include all the mitosporic Trichocomaceae (Eurotiomycetes) except *Penicillium marneffei*, *Fusarium oxysporum* and *Magnaporthe oryzae* (Sordariomycetes), *Candida dubliensis* and *Schizosaccharomyces pombe* (Saccharomycetes) and *Filobasidiella neoformans* (Basidiomycota) (R. te Biesebeke, private communication).

The Supplemental Data Figs. S1 and S2 provide the MAFFT alignments of representative F and S globin sequences, respectively. The sequences are identified by the first three letters of the binary species name, the number of residues (if there are more than a single sequence), and the first three or four letters of the phylum, followed by the first three letters of the family (see Table S1).

### Molecular phylogeny of fungal F globins

**Figure 3:**
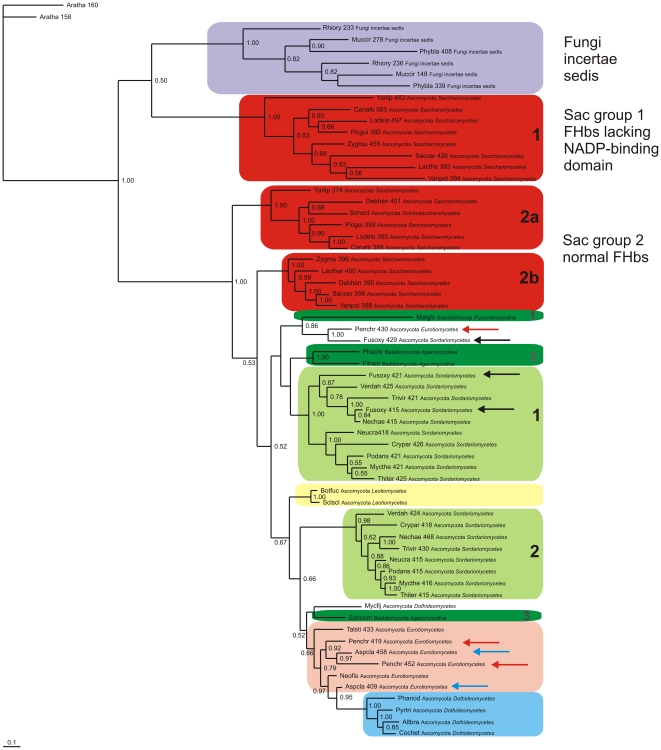
Bayesian phylogenetic tree of fungal FHbs. Bayesian tree based on a MAFFT v.6.850 alignment of 62 fungal FHb globin domains using two plant nonsymbiotic Hbs as outgroup. Support values at branches represent Bayesian posterior probabilities (>0.5). The sequences are identified by the first three letters of the binary species name, the number of residues, and the full phylum and family names (see Table S1). Sac – Saccharomycetes.

The MAFFT alignment of 62 representative FHb globin domains selected from over 130 sequences was found to have the highest MUMSA score of the five alignments we tried (Supplemental Data Table S2). Fig. 3 shows the resulting Bayesian phylogenetic tree obtained using two *A. thaliana* nonsymbiotic plant Hbs (NsHbs) as outgroup. The Fungi incertae sedis F globins (purple box) cluster separately from the remaining fungal F globins. Closest to them are the Saccharomycete group 1 chimeric FHbs (red) lacking the C-terminal moiety of the reductase domain (Fig. 2). The normal FHbs of the Saccharomycetes are represented by groups 2a and 2b (red boxes). Most of the Sordariomycetes (Ascomycota) have at least two normal FHbs, which again cluster in two groups, (light green boxes 1 and 2) in Fig. 3. Although most of the Eurotiomycetes have two or more FHbs, they cluster together, as exemplified by the two *Aspergillus clavatus* FHbs (blue arrows). The Sordariomycete *Fusarium oxysporum* has three FHbs (black arrows) that cluster closely together, in contrast to the three *Penicillium chrysogenum* FHbs (red arrows) representing Eurotiomycetes. The Dothideomycetes (blue box) and the two Leotiomycetes (yellow box) have single FHbs, as do all the Basidiomycota (green boxes). The latter four sequences are split into three separate groups: one and two (green boxes 1 and 2) encompassing a eurotiomycete and a sordariomycete sequence, while the fourth one (green box 3) lies with a dothideomycete FHb.

A Bayesian tree of the same FHbs as in Fig. 3, employing the complete (globin+reductase) sequences and omitting the Fungi incertae sedis and the two outgroup sequences, is shown in the Supplemental Data Fig. S3. The topologies of the two trees are similar, with almost complete preservation of all the clusters except for their relative locations in the tree. Note in particular that the Saccharomyces group 2 sequences form two separate clusters 2a and 2b in both trees. The topologies of the corresponding RAxML trees (not shown) are in agreement with the Bayesian trees.

### Molecular phylogeny of fungal and bacterial FHbs

**Figure 4:**
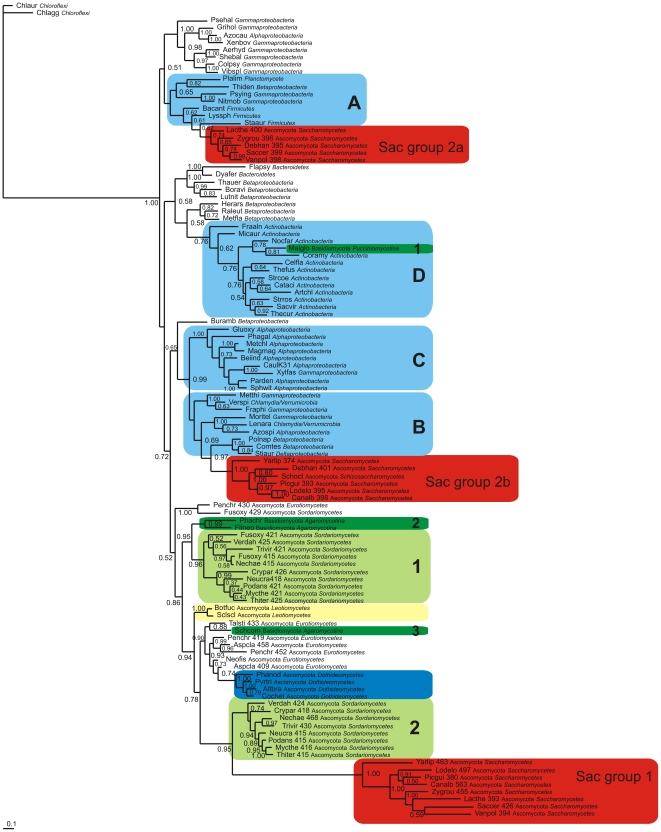
Bayesian phylogenetic tree of fungal and bacterial FHbs. Bayesian tree based on a T-COFFEE 9.01 alignment of the globin domains of 55 representative fungal FHbs and 54 representative bacterial FHbs. Support values at branches represent Bayesian posterior probabilities (>0.5). All the bacterial FHbs are in the blue boxes. The sequences are identified by the first three letters of the binary species name, the number of residues, and the full phylum and family names (see Table S1). Sac – Saccharomycetes.

In contrast to Fungi, where with the exception of the Fungi Incertae sedis, all F globins are chimeric FHbs, bacterial F globins comprise FHbs and single domain Fgbs [15]. We used the globin domains of 55 representative bacterial FHbs selected from over 300 bacterial FHbs [15], to align with the globin domains of 37 representative fungal FHbs culled from the 62 sequences used in Fig. 3. Fig. 4 shows a Bayesian tree based on the TCOFFEE v.9.01 alignment, that provided the highest MUMSA score (Supplemental Data Table S2) using two bacterial protoglobins (Pgbs) as outgroup. Although the majority of the fungal FHbs, including the Sordariomycete FHbs (light green boxes 1 and 2) and three of the four Basidiomycota FHbs (green boxes 2 and 3) cluster together, the Saccharomycete group 2 FHbs are split in two groups (as in Fig. 3) surrounded by bacterial sequences. The Saccharomycete group 2a clusters with 3 Proteobacteria, 3 Bacilli (Firmicutes) and a Planctomycete (blue box A). The Saccharomycete group 2b cluster next to 7 Proteobacteria and 2 Chlamydia/Verrumicrobia (blue box B). The fourth Basidiomycota (green box 1) is embedded within 12 Actinobacteria (blue box D). The Neighbor Joining (NJ) tree provided in Supplemental Data Fig. S4 supports the Bayesian results. The Saccharomycete group 2 sequences are again split into separate clusters. Furthermore, the Saccharomycete group 2a FHbs cluster with the bacterial cluster A (same as in Fig. 4, except for a Planctomycete FHb) and the group 2b is vicinal to bacterial cluster B (same as in Fig. 4). The 4 Basidiomycota sequences are again split into three groups, with the *Malassezia globosa* FHb (green box 1) surrounded by the same 12 Actinomycete sequences as in Fig. 4 (blue box D).

### Molecular phylogeny of fungal S globins

**Figure 5:**
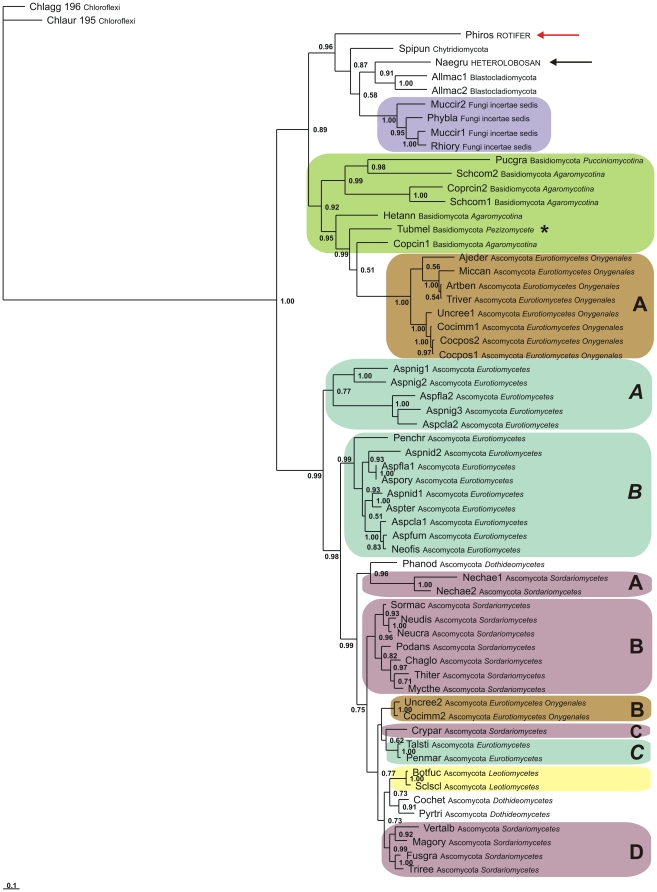
Bayesian phylogenetic tree of fungal Sgbs. Bayesian tree based on a MAFFT v.6.850 alignment of 59 fungal, one rotifer and one heterolobosan Sgbs, using two bacterial Pgbs as outgroup. Support values at branches represent Bayesian posterior probabilities (>0.5). The sequences are identified by the first three letters of the binary species name, and the full phylum and family names (see Table S1).

In addition to over 70 fungal Sgbs, our *in silico* searches revealed the presence of two previously unknown eukaryote Sgbs, from the rotifer *Philodina roseola* (ACD54784.1) and the heterolobosan *Naegleri gruberi* (XP_002677420.1). They represent the only other eukaryote Sgbs known at present and were added to the fungal sequences. In the Bayesian tree based on a MAFFT v.6.850 alignment shown in Fig. 5, the rotifer and heterolobosan Sgbs are identified by red and black arrows, respectively. The tree demonstrates with good support, that the fungal Sgbs separate into two distinct groups. One, consists of two well-separated clades, one comprising the two nonfungal Sgbs, the Chytridiomycota, the Blastocladiomycota and the Fungi incertae sedis (purple) Sgbs, and the other encompassing the Basidiomycota (green), including the lone Pezizomycota *Tuber melanosporum* (star), and most of the Onygenales, a subgroup of the Eurotiomycetes (light brown box A), that appear to be unique in having no FHbs (see Table S1). The second group comprises the remaining Ascomycota Sgbs, including the second sequences of the two Onygenales that have two Sgbs, *Uncinocarpus reeseii* and *Coccidioides immitis* (light brown box B). Note that the Eurotiomycetes (light blue boxes), Sordariomycetes (light purple boxes) and Dothideomycetes, all cluster into clades, 3 (light blue A–C), 4 (light purple boxes A–D) and 2, respectively. The NJ tree based on a TCOFFEE v.9.01 alignment, provided in Supplemental Data Fig. S5 shows the same clustering of Sgb sequences, except for their relative tree locations. The Bayesian tree based on the TCOFFEE v.9.01 alignment is shown in Fig. S6.

### Molecular phylogeny of fungal and bacterial S globins

Within bacteria, the S globins occur predominantly as a variety of globin-coupled sensors (GCSs), many with more than a single non-globin domain, and two distinct types of single domain globins, the protoglobins (Pgbs) and the Sgbs [15]. Figs. S7 and S8 show a Bayesian and NJ trees based on a MUSCLE alignment of the globin domains of 60 representative bacterial S globins out of over 425 [15], of which six were Pgbs (blue box) (including two Archaea) and five were Sgbs (light brown boxes). Interestingly, the GCSs clustered in separate groups, three comprising diguanylate cyclases (D, yellow boxes), three consisting of methyl accepting chemotaxis proteins (M, red boxes), one histidine kinase (H, light blue), two Sulfate Transporter and AntiSigma factor antagonist (STAS) domain proteins (S, purple) and five unknown domain proteins (U, green). The D* group consists of diguanylate cyclases with additional domains. The U and S sequences have about 300 residues and are the smallest of the S chimeric globins, and may well be related to the fungal Sgbs.

**Figure 6:**
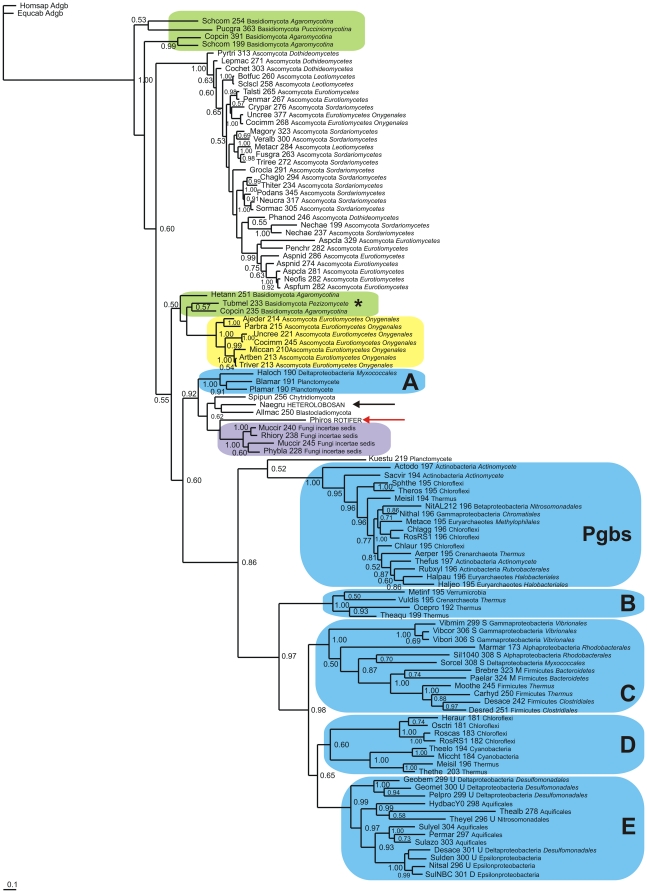
Bayesian phylogenetic tree of fungal and bacterial Sgbs. Bayesian tree based on a T-COFFEE 9.01 alignment of 51 fungal, 57 bacterial (including 16 Pgbs), one rotifer and one heterolobosan Sgbs, using two Adgb sequences [16] as outgroup. Support values at branches represent Bayesian posterior probabilities (>0.5). The sequences are identified by the first three letters of the binary species name, the number of residues, and the full phylum and family names (see Table S1).

Next, we selected 40 representative bacterial Sgbs, consisting of 16 Pgbs (including 5 Archaea), and Sgbs with less than 320 residues. The results of a Bayesian analysis based on a TCOFFEE v. 9.01 alignment (highest MUMSA score of the 5 alignments, see Table S2) of the bacterial Sgbs with 51 fungal, one rotifer and one heterolobosan Sgbs, using two androglobin (Adgb) sequences [16] as outgroup, are shown in Fig. 6. All the bacterial Sgbs (light blue) cluster away from the eukaryote Sgbs, except for 3 sequences (blue box A) intercalated between the Fungi incertae sedis Sgbs (purple) and the four sequences comprising the rotifer (red arrow), heterolobosan (black arrow), Blastocladiomycete and Chytridomycete Sgbs. The clustering observed in Fig. 6 is also present in the corresponding NJ tree shown in Fig. S9.

### Molecular phylogeny of T globins

**Figure 7:**
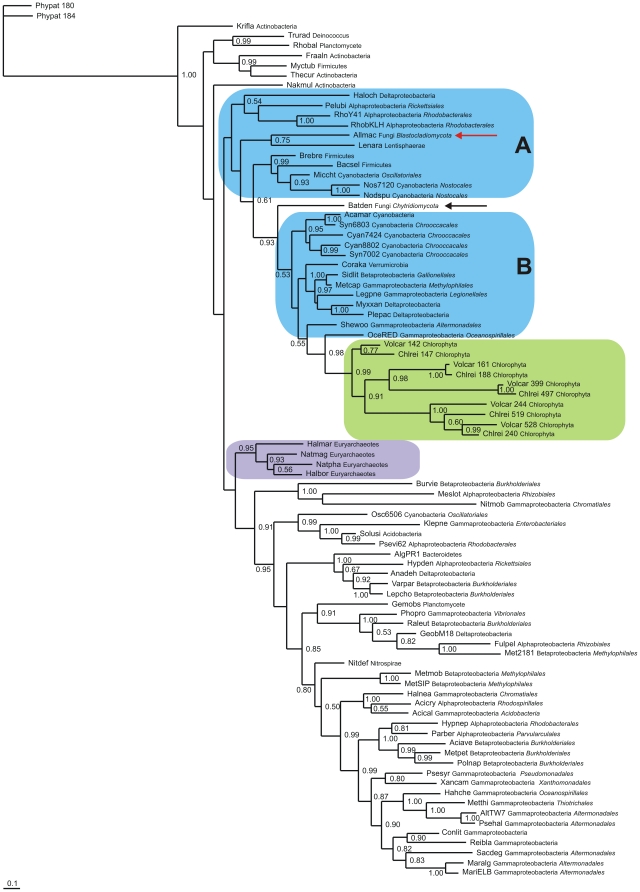
Bayesian phylogenetic tree of fungal and bacterial T1 globins. Bayesian tree based on a T-COFFEE 9.01 alignment of 2 fungal (red and black arrows), 70 bacterial (blue), 4 euryarchaeote (purple) and 10 chlorophyte (green) T1 globins, using 2 *Physcomitrella* nsHbs as outgroup. Support values at branches represent Bayesian posterior probabilities (>0.5). All the bacterial FHbs are in the blue boxes. The sequences are identified by the first three letters of the binary species name, the number of residues, and the full phylum and family names (see Table S1).

We also investigated the phylogeny of the newly identified T globins group 1 (T1 globins) of *Allomyces macrogynus* (Blastocladiomycota) and *Batrachochytrium dendrobatidis* (Chytridiomycota). Fig. 7 presents the results of a Bayesian analysis based on a TCOFFEE v. 9.01 alignment of the two T1 globins (red and black arrows) with 74 bacterial sequences selected from over 150 known sequences [15], including 4 Euryarchaeota sequences (purple), that provided the highest MUMSA score (Table S2), and employing 2 plant globins (*Physcomitrella patens* NsHbs) as outgroup. We also added 10 chlorophyte T1 globins (green), as additional representative microbial eukaryote T1 globins. The Blastocladiomycota T1 sequence (red arrow) clusters with a Lentisphaera, 3 Alphaproteobacteria and 3 Cyanobacteria T1s (blue box A). Next to this cluster lies the Chytridiomycote T1 (black arrow), vicinal to a group comprising 5 Cyanobacteria and 7 Proteobacteria (blue box B). Adjacent is the clade of the 10 Chlorophyte sequences (green box). In the corresponding NJ tree, provided in Fig. S10 the same clustering is observed except for the Blastocladiomycota T1 globin.

## Discussion

### An overview of fungal globins

Because the taxonomy of fungi is still in a state of flux, we used the GenBank classification (www.ncbi.nlm.nih.gov/Taxonomy) in Fig. 1 and Supplemental Data Table S1, which follows recent recommendations [17], [18], [19]. The phylum Zygomycota is no longer recognized, and its members are now divided among the new phylum Glomeromycota and four Fungi incertae sedis subphyla, including the Mucoromycotina. Furthermore, the Microsporidia, unicellular animal parasites with highly reduced genomes were added as a new phylum [17], [18], [20]. The most recent trees of fungi [21], [22] are in agreement with the proposed phylogeny. The estimates of the numbers of species present in the major divisions are taken from the *Dictionary of the Fungi* (10th edition) [23]. Although the total number of fungal species now stands at about 100'000, several estimates suggest a much higher number, perhaps as much as ten-fold higher [24], [25].

The sequences of over 165 fungal genomes accumulated subsequent to the landmark sequencing of the genome from the yeast *Saccharomyces cerevisiae* some 15 years ago, has allowed us to perform a census of putative globins and to obtain a sufficient coverage to generate a comprehensive view of their distribution listed in Supplemental Data Table S1 and summarized in Fig. 1. Overall, we were able to detect at least one authentic globin sequence in the genome assemblies of over 90% of Ascomycota and about 60% of Basidiomycota species. The F globins are limited to FHbs in all fungi, except the Fungi incertae sedis, where they are single domain Fgbs (see Table S1). In contrast, the fungal sensor globins, unlike the bacterial S family, are limited to single domain Sgbs. It should be pointed out that since less than half of the fungal genomes have been completed, it is not always possible to be sure whether globins are completely absent in a given genome, or all possible globins are accounted for. The numerically dominant Ascomycota appear to have globins in all their subgroups except the Pneumocystidomycetes (Fig. 1). Overall, the Basidiomycota, have a much lower percent of genomes with globins, particularly among the Pucciniomycotina, with only one of six genomes to have a Sgb and no F globins. The Glomeromycota, known as arbuscular mycorrhizae fungi, form symbiotic associations with the roots of over 80% of vascular plant families [26]; the only genome representing them appears to have no globins. The Neocallimastomycota are anaerobic fungi that reside mostly in the stomachs of ruminants [27]; no genome has been sequenced. Among the Fungi incertae sedis, all three Mucoromycotina genomes have globins. Finally, the Microsporidia, obligate intracellular parasites with genomes that are 5-fold to >20-fold reduced genomes relative to other fungi and which relies on ATP import from its host [28], are represented by seven genomes, none of which have globins.

The finding of group 1 T globins in *Allomyces macrogynus* (Blastocladiomycota) and *Batrachochytrium dendrobatidis* (Chytridiomycota) was an unexpected byproduct of our census. In the former, the T globin domain occurs in the C-terminal moiety (826–944) of a 1129 residue chimeric protein. A CDD search [29] revealed a leucine-rich repeat containing domain (578–800) comprising several 11-residue segments LxxLxLxxN/CxL, that are conserved in LRR proteins [30]. Although the C-terminal 185 residues remain unidentified, the N-terminal (20–560) exhibits a high Z = 30.5 in a FUGUE search [31] with a composite profile of the ribonuclease inhibitors from *Sus scrofa, Homo sapiens* and *Schizosaccharomyces pombe* (PDB: 2bnh, 1a4y, 1yrg, respectively). *Batrachochytrium dendrobatidis*, the causative agent of chytridiomycosis in amphibians [32], has only one globin, a single domain 116 residue T1.

### Molecular phylogenetic analyses

The results of our Bayesian, RAxML and NJ analyses presented in the Results section clearly require special discussion. They were performed on the one alignment out of five that had the highest MUMSA score. Furthermore, we used two globin sequences, selected *ad hoc*, and different from the fungal globins as outgroup. The main weakness of all the phylogenetic trees we show consists in the low node probabilities. Nevertheless, despite the low posterior probabilities and bootstrap values, too low to provide unequivocal phylogenies, the trees obtained by the Bayesian, RAxML and NJ analyses show mostly broad agreement with each other. In particular, the specific clusters of sequences are generally conserved, even though their tree locations vary, depending on the alignment used and the type of molecular phylogenetic analysis employed.

Earlier, we put forward a model of globin evolution [14] based on the known distribution of globins in the three kingdoms of life. Since Bacteria are the only kingdom to have all the representatives of the F, S and T globin families, we proposed that Eukaryote globins were derived from the appropriate bacterial lineage via HGT as the result of one or both of the accepted endosymbiotic events responsible for the origin of mitochondria and chloroplastids, involving an α-proteobacterium and a cyanobacterium respectively [15]. Our second aim in this study was to determine whether the fungal FHbs, Sgbs and T1 globins could have originated in a manner compatible with the proposed model. Thus, although the phylogenetic trees that we obtained are far from being reliable, we feel that the persistent clustering of specific sequences observed in the trees based on the Bayesian, ML and NJ analyses allows us to draw some tentative conclusions.

Another aspect of our analyses that requires elaboration, is the selection of representative sequences, fungal on one side and bacterial on the other, necessitated by the large number of available sequences. The selection was based on initial NJ phylogenetic trees based on multiple sequence alignments generated by MAFFT 6.833. In all cases we selected the sequences to maintain representation of the distinct groups, so that e.g. in bacterial FHbs, we included representatives of the major bacterial phyla, such as Actinobacteria, Bacteroidetes, Chlorobi, Deinococcus, Firmicutes (Bacilli) and the five subdivisions of the Proteobacteria in approximate proportion to the actual number of FHbs known.

### Molecular phylogeny of fungal F globins

The results of Bayesian analyses based on the alignments of the globin domain only (Fig. 3) and of the complete FHb sequences (Fig. S3), reveal an unexpected complexity in the phylogeny of Dikarya globins. First, the multiple FHbs of the Saccharomycetes (red boxes 1 and 2) occur as widely separated clusters. Furthermore, cluster 2 is separated into neighboring clusters 2a and 2b, similar to the the Sordariomycete globins (light green boxes 1 and 2). The Saccharomycete group 1 FHbs, widely divergent from the remaining FHbs, represent a new group of FHbs, wherein an unidentified protein is covalently linked to a FHb missing its cqx3 domain (see Fig. 2). The Eurotiomycetes, Dothideomycetes and Leotiomycetes have mostly single FHbs and cluster separately (light brown, light purple and yellow, respectively). Second, the Basidiomycota FHbs (dark green boxes) do not cluster separately from the Ascomycota FHbs as would be expected on the basis of the overall fungal phylogeny shown in Fig. 1. Instead, they cluster in three groups (green boxes 1–3). The first two are close to some of the Sordariomycetes (light green box 1) and Eurotiomycetes (light brown box), the third one is next to a Dothideomycete.

Examination of Table S1 demonstrates that among the numerically preponderant Ascomycota, the Sordariomycetes and Saccharomycetes have two to three FHbs. It is recognized that the emergence of Saccharomycetes was preceded by a whole genome duplication (WGD), followed by gene loss, diversification and segmental gene duplication (SGD) [33], [34], [35], [36]. Thus, while the two groups of Saccharomycete FHbs could well be due to WGD followed by neo- or sub-functionalization, additional FHbs in e.g. the *Candida* species, and abnormal FHbs, e.g. in *K. waltii* (Table S1), would occur via single SGD. In the case of *A. gossypii*, there must have occurred a loss of the normal FHb.

Since no WGD is recognized to have occurred in Sordariomycetes, there the two FHb lineages must have occurred via SGD in their ancestor. Furthermore, several Sordariomycetes have an additional FHb-like protein, wherein a globin domain is fused to an unknown C-terminal domain (Table S1).

Both the Dothideomycetes and Eurotiomycetes appear to cluster as independent clades in the trees shown in Fig. 3 and Fig. S3. Although the former have only single FHbs, the latter may have up to three different FHbs, e.g. *P. chrysogenum* (red arrows). Although the *Aspergillus sp.* have two FHbs, they appear to be closely related (blue arrows). Te Biesebeke and his collaborators have recently examined the phylogeny of *Aspergillus sp.* FHbs as well as several related fungi [37]. Their phylogenetic tree shows that there are two distinct clades of *Aspergillus* FHbs, one being closer to the Dothideomycetes, and the other to the Basidiomycetes.

### Relationship of fungal to bacterial FHbs

To investigate the relationship of fungal to bacterial FHbs, we carried out Bayesian and NJ analyses based on a TCOFFEE v.9.01 alignment of representative sets of fungal and bacterial FHbs in Fig. 4 and Fig. S4, respectively. Both trees reproduce the clustering of the Saccharomycete group 1, 2a and 2b sequences and of the four Basidiomycota globins. Of the Ascomycota FHbs, only the group 2a and 2b cluster with bacterial FHbs, the former with 2 Bacilli (Firmicutes), 3 Proteobacteria and one Planctomycete (blue box A), and the latter vicinal to 7 Proteobacteria and 2 Chlamydia/Verrumicrobia (blue box B). Of the four Basidiomycota FHbs, only the *Malassezia globosa* FHb nestles within 11 Actinomycete FHbs, These results points to the possibility of HGT events in fungal phylogeny. Within the last several years, many instances of HGT from prokaryotes to fungi [38], fungi to fungi [39], [40], [41] and plants to fungi [42] have come to light.

The complexity of bacterial taxonomy can be reduced by viewing the bacterial taxa at a lower resolution. All the diversity of prokaryotes is encompassed in 37 taxa; these can be grouped into five super taxa: the Archaea, the Actinobacteria, the Clostridia, the Bacilli and the Double Membrane Prokaryotes (DMPs) [43]. The latter comprise 21 of the 37 taxa, including Acidobacteria, Aquificae, Bacteroidetes, Chloroflexi, Chlorobi, Chlamidiae, Cyanobacteria, Deinococcus-Thermus, Proteobacteria, Planctomyces and Verrumicrobia. Thus, the Ascomycota group 2a FHbs occur next to Bacilli and DMPs, the group 2b are vicinal to DMPs and only the *M. globosa* FHb occurs within a clade of Actinomycete FHbs.

The intermixing of fungal and bacterial FHbs is underscored by the results of PSIBLAST searches using *Aspergillus clavatus* (Ascomycota) FHb globin domain shown in Supplementary Data Table S3. In this particular case, apart from other fungal FHbs, the hits are dominated by Proteobacterial FHbs. Note also the hits on eukaryote 3/3 Hbs, including vertebrate Ngbs, in agreement with the proposed membership of all metazoan globins in the bacterial F globin family [7], [8], [15].

### Fungal Sgbs and their relationship to bacterial Sgbs

In Fig. 5, we show a Bayesian tree based on a MAFFT v.6.933 alignment of 59 fungal, one bdelloid Rotifer *Philodina* (“wheel-bearer”) (red arrow) and one amebo-flagellate *Naegleria* (Heterolobosan) (black arrow) Sgbs. We used the MAFFT alignment even though its MUMSA score was less than the TCOFFEE v.9.01 alignment (Table S2), because the Bayesian tree based on the latter (Fig. S6) shows unexpectedly the Eurotiomycete family Onygenales as the unlikely deep early branch. The topology in Fig. 5 is similar to that of NJ tree based on the TCOFFEE v.9.01 alignment in Supplementary Data Fig. S5.

Fig. 6 shows a Bayesian tree based on a TCOFFEE v.9.01 alignment of representative fungal and bacterial Sgbs. Its topology is similar to the corresponding NJ tree in Supplementary Data Fig. S9. In these trees the *Philodina* (red arrow) and *Naegleria* (black arrow) Sgbs cluster with the Blastochladiomycota and Chytridiomycota Sgbs and with the Fungi incertae sedis (purple) separated by a Proteobacteria and 2 Planctomycete sequences (blue box A). The similarity of the planctomycete to the fungal Sgbs is underscored by the results of BLASTP searches provided in Supplemental Data Tables S4 and S5. All these results suggest that the fungal and the two other eukaryote Sgbs share a common ancestry with each other and the bacterial Planctomycetes.

### Relationship of fungal T1 globins to bacterial T1 globins

We included 10 Chlorophyte sequences in our analysis of the phylogenetic relationship between the fungal and the bacterial T1s, because we have previously investigated their phylogeny [44]. The two fungal sequences occur in separate clades in the Bayesian tree shown in Fig. 7, the Blastocldiomycota T1 clustering with Proteobacteria, Cyanobacteria and Bacilli (Firmicutes) (blue box A). The Chytridiomycota T1 clusters with Cyanobacteria and Proteobacteria (blue box B), vicinal to the Chlorophyte sequences (*Volvox* and *Chlamydomonas*) (green box). The bacterial T1s clustering with the fungal, Euryarchaea and Chlorophyte sequences include all the bacterial T1s that clustered with the 20 Chlorophyte T1s (see Fig. 4 in reference [44]). This result, together with the results of BLASTP searches provided in Supplemental Data Tables S6 and S7, are again consonant with the proposed model of globin evolution.

### Possible functions of fungal globins

The Fungi exhibit osmotrophic growth, wherein the required nutrients are produced via the breakdown of substrates by secreted extracellular enzymes. Furthermore, they evince two distinct morphologies: unicellular, in which growth occurs via budding or simple fission and multicellular filamentous, wherein growth proceeds via production of hyphal strands that aggregate to form a mycelium. The osmotrophic growth capability is a very effective tool for the colonization of diverse habitats and has allowed the fungi to play a dominant role in biomass degradation in terrestrial ecosystems [45] and to also become important plant and animal pathogens. The fungi are one of the most ancient eukaryote families and it is estimated that the Saccharomycotina (budding yeasts) and the Pezizomycotina (filamentous ascomycetes) have diverged from one another some 900–1000 million years ago (Mya) [46]. Furthermore, the extent of divergence within the Saccharomycotina alone is greater than the Chordate phylum of the animal kingdom [47].

A number of studies over the last decade have demonstrated the FHbs to play a role in the response of pathogenic fungi to nitric oxide, the antipathogenic compound produced by the innate immune system [48]. The FHbs protect against the toxic effects of nitric oxide via their NO dioxygenase activity that converts NO to nitrate, discovered by P. Gardner and his collaborators [49]. Additional information became recently available regarding the FHbs of *Aspergillus oryzae* and *A. niger* (Eurotiomycetes). They both have two FHbs, FHb1 (416/417 residues) and FHb2 (436/439 residues) [37], [50], [51], in agreement with our findings; moreover, the FHb2's N-terminals contain a probable signal sequence. Shoun and his collaborators have shown that the *A. oryzae* FHb1 and FHb2 localize to the cytosol and the mitochondria, respectively [51]. FHb1 is monomeric and can use either NADH or NADPH as electron donor, while FHb2 is dimeric and can use only NADH [50]. Interestingly, only the expression of the cytosolic FHb1 is upregulated in the presence of NO. It was also suggested that the reductases of the two FHbs tend to promote oxidative damage [52]. Te Biesebeke and his collaborators have shown that the transcription levels of the foregoing *Aspergillus* species FHb1s appears to be positively correlated with hyphal growth [37]. Furthermore, not all the N-terminal extensions have predicted mitochondrial transit peptides as observed for *A. nidulans*, *A. terreus*, *A. fumigatus* and *A. oryzae* [37] but rather have predicted signal peptides suggesting that localization of the FHbs is species dependent. In the case of the saccharomycete *Candida albicans*, the most prevalent human fungal pathogen, the response to nitric oxide is complex, accompanied by overexpression of many genes, including only one of the several FHb genes [53], [54]. We conclude this very brief overview of FHb function in fungi, by pointing out that the NO dioxygenase function is not unique to FHbs: although all globins can carry out this reaction, carrying it out repeatedly, requires the added presence of a reductase [55], [56], [57].

Nothing is known about the structure and function of fungal Sgbs. All we can do is make inferences based on recent results obtained with other members of the bacterial S family. One, is the recent finding by Alam and his collaborators that a Pgb fused to the MCP signaling domain of the *E. coli* chemotaxis transducer Tsr, was also able to bind O_2_ reversibly and elicit an aerotactic response similar to the wild type GCSs [58]. Another, is the inference that hexacoordination may also occur in fungal Sgbs, based on the recent crystal structure of the globin domain of *Geobacter sulfurreducens* (Deltaproteobacteria) GCS, which found bis-histidyl hexacoordination via HisE11 and HisF8 [59]. More recently, Gillez-Gonzalez and her collaborators have demonstrated a role for GCSs in the synthesis and control of c-di-GMP in bacteria [60], [61].

### Conclusion

This census of putative globin sequences in the Fungi has emphasized the broad presence of globins from the F and S families. We have identified globins in 136 out of 165 available genomes including a novel class of FHbs lacking the C-terminal portion of the reductase moiety as well as 2 previously unidentified T1s. Molecular phylogenetic analyses revealed a complex evolutionary relationship between fungal and bacterial globins. Our results, summarized in Table 1, list the present day bacterial phyla whose FHbs and Sgbs may share ancestry with the fungal FHbs and Sgbs. The results agree with our model of globin evolution [14], except in the case of the single Pucciniomyceta (Basidiomycota) FHb that appears to be related to Actinomycete FHbs. Furthermore, they clearly do not rule out the possibility of the occurrence of multiple HGT events leading to the appearance of present day fungal globins.

**Table 1:** Bacterial phyla whose globins may share ancestry with fungal globins.

Globins	Possible bacterial ancestor
Saccharomycete group 2a FHbs	Bacilli (Firmicutes), Proteobacteria, Planctomycetes
Saccharomycete group 2b FHbs	Proteobacteria, Chlamydia/Verrumicrobia
Basidiomycota, Pucciniomycotina FHb	Actinomycetes (Actinobacteria)
Fungi, Rotifer, Heterolobosan Sgbs	Planctomycetes, Proteobacteria
Blastocladiomycota T1 gb	Cyanobacteria, Proteobacteria
Chytridiomycota, Chlorophyte T1 gbs	Cyanobacteria, Proteobacteria

## Methods

### Identification of globin sequences

Putative globins and globin domains were identified from the SUPERFAMILY globin gene assignments (http://supfam.mrc-lmb.cam.ac.uk) [62], and via BLASTP [63] and PSIBLAST [64] searches of the GenBank non-redundant database, BLASTP and TBLASTN searches of fungal genomes (http://blast.ncbi.nlm.nih.gov//sutils/genom_table.cgi?organism=fungi), and WU-BLAST2 searches (http://www.yeastgenome.org/cgi-bin/blast-fungal.pl). All the sequences were subjected to a FUGUE search [31] (http://www-cryst.bioc.cam.ac.uk). Given a query sequence, FUGUE scans a database of structural profiles, calculates the sequence-structure compatibility scores for each entry, using environment-specific substitution tables and structure-dependent gap penalties, and produces a list of potential homologs and alignments. FUGUE assesses the similarity between the query and a given structure via the Z score, the number of standard deviations above the mean score obtained by chance: the default threshold Z = 6.0 corresponds to 99% probability [31].

### Sequence alignment and myoglobin-fold criteria

Multiple sequence alignments were obtained using the following algorithms: TCOFFEE v.9.01 [65] (http://www.tcoffee.org), PROBCONS [66] (http://toolkit.tuebingen.mpg.de/probcons), COBALT [67] (www.ncbi.nlm.nih.gov/tools/cobalt/), and MAFFT 6.833 [68], [69], and MUSCLE 3.7 [70] available at EMBL-EBI (www.ebi.ac.uk/Tools/). The resulting alignments were checked manually for the conservation of the F8 His, and their quality was assessed using MUMSA [71], [72] (http://msa.cgb.ki.se).

All known globin sequences exhibit the Mb-fold [73], [74], the pattern of predominantly hydrophobic residues at 36 conserved, solvent-inaccessible positions, including 33 intra-helical residues defining helices A through H (A8, A11–12, A15, B6, B9–10, B13–14, C4, E4, E7–8, E11–12, E15, E18–19, F1, F4, G5, G8, G11–12–13, G15–16, H7–8, H11–12, H15, and H19), the two inter-helical residues at CD1 and FG4, and the invariant His at F8. Our criteria for a satisfactory Mb-fold required a FUGUE Z score >6 and a His at the proximal F8 position.

It is necessary to point out that since FHbs are chimeric proteins, and since they and the Sgbs have substantial N- and C-terminal extensions, setting the boundaries of the globin domains was an arbitrary decision. After one round of alignment, the sequences were trimmed, assuming the globin domain to start 11 residues prior to the B10 position and to end 15 residues after the H8 position. The trimmed sequences were then used in the final alignments.

### Molecular phylogeny

A total of over 130 F globin sequences, including about 30 of the chimeric incomplete FHbs and over 70 S globin sequences were identified. These were aligned using PROBCONS, MUSCLE v.3.7, MAFFT v.6.850, COBALT and TCOFFEE v.9.01. The quality of the alignments was assessed by MUMSA and the two top scoring alignments were subjected to Bayesian, Maximum Likelihood (ML) and Neighbor-Joining (NJ) phylogenetic analyses.

Bayesian inference trees were obtained employing MrBayes version 3.1.2 [75], using the WAG model of amino acid evolution [76] and assuming a gamma distribution of evolution rates, as indicated by analysis of the alignment using ProtTest [77]. Two parallel runs, each consisting of four chains were run simultaneously for up to 6×10^6^ generations. Trees were sampled every 100 generations, and the burnin value was set to 2×10^4^. In all analyses convergence of the two parallel runs was observed. Maximum likelihood-based phylogenetic analysis was performed by RAxML version 7.2.3 [78] assuming the WAG model and gamma distribution of substitution rates. The resulting tree was tested by bootstrapping with 100 replicates. Neighbor-joining analyses were performed using MEGA version 5.0 [79]. Distances were corrected for superimposed events using the Poisson method. All positions containing alignment gaps and missing data were eliminated only in pairwise sequence comparisons (pairwise deletion option). The reliability of the branching pattern was tested by bootstrap analysis with 1000 replications.

## Supporting Information

Figure S1 **MAFFT alignments of fungal FHbs.**
(TIF)

Figure S2 **MAFFT alignments of fungal Sgbs.**
(TIF)

Figure S3 **Bayesian phylogenetic tree of complete fungal FHbs.** Bayesian tree of a MAFFT v.6.850 alignment of 62 complete (globin+reductase domains) fungal FHbs, including all the sequences in Fig. 3, except the Fungi incertae sedis and the two outgroup sequences. Support values at branches represent Bayesian posterior probabilities (>0.5). The sequences are identified by the first three letters of the binary species name, the number of residues, and the full phylum and family names (see Table S1).
(TIF)

Figure S4 **NJ phylogenetic tree of fungal and bacterial FHbs.** NJ tree of a TCOFFEE v. 9.01 alignment of 37 representative fungal FHbs and 55 representative bacterial FHbs using two bacterial Pgbs as outgroup. Only bootstrap values >50% are shown. The sequences are identified by the first three letters of the binary species name, the number of residues, and the first three or four letters of the phylum, followed by the first three letters of the family (see Table S1). Abbreviations: ASC – Ascomycota; Dot – Dothideomycetes; Eur – Eurotiomycetes; Leo – Leotiomycetes; Sac – Saccharomycetes; Sor – Sordariomycetes; BAS – Basidiomycota; Aga – Agaromycotina; Puc – Pucciniomycotina; ACT – Actinobacteria; APR – Alphaproteobacteria; BPR – Betaproteobacteria; GPR – Gammaproteobacteria; DPR – Deltaproteobacteria; BAC – Bacteroidetes; CHL – Chlamydia/Verrumicrobia; FIR – Firmicutes; PLA – Planctomycete.
(TIF)

Figure S5 **NJ phylogenetic tree of fungal Sgbs.** NJ tree of a TCOFFEE v. 9.01 alignment of 59 fungal, one rotifer and one heterolobosan Sgbs. Only bootstrap values >50% are shown. The sequences are identified by the first three letters of the binary species name, the number of residues, and the first three or four letters of the phylum, followed by the first three letters of the family (see Table S1). Abbreviations: FINSE - Fungi incertae sedis; ASC – Ascomycota; Dot – Dothideomycetes; Eur – Eurotiomycetes; Pez – Pezizomycete; Sac – Saccharomycetes; Sor – Sordariomycetes; BAS – Basidiomycota; Aga – Agaromycotina; Puc – Pucciniomycotina; BLA – Blastocladiomycota; CHLO – Chloroflexi; CHY – Chytridiomycota; HETER – Heterolobosan; ONY – Onygenales; PEZ – Pezizomycotina; ROTI - Rotifer.
(TIF)

Figure S6 **Bayesian phylogenetic tree of fungal Sgbs.** Bayesian tree based on a TCOFFEE v. 9.01 alignment of 59 fungal, one rotifer and one heterolobosan Sgbs. Support values at branches represent Bayesian posterior probabilities (>0.5). The sequences are identified by the first three letters of the binary species name, the number of residues, and the full phylum and family names (see Table S1).
(TIF)

Figure S7 **Bayesian phylogenetic tree of bacterial GCSs and Sgbs.** Bayesian tree based on a MUSCLE v.6.850 alignment of 60 representative bacterial GCSs including 5 single domain sensor globins (light brown boxes). Support values at branches represent Bayesian posterior probabilities (>0.5). The first three letters of each part of the binary species name is followed by the number of residues and by a letter denoting the nonglobin domain: D – diguanylate cyclase; D* - diguanylate cyclase with additional domains; M – methyl accepting chemotaxis domain; H – histidine kinase domain; S – STAS domain; Pgb – protoglobin; Sgb – single domain sensor globin; U – unidentified domain.
(TIF)

Figure S8 **NJ phylogenetic tree of bacterial GCSs and Sgbs.** NJ tree of a MUSCLE v.6.850 alignment of 60 representative bacterial GCSs including 5 single domain sensor globins (light brown boxes). Only bootstrap values >50% are shown. The first three letters of each part of the binary is followed by number of residues and by a letter denoting the nonglobin domain: D – diguanylate cyclase; D* - diguanylate cyclase with additional domains; M – methyl accepting chemotaxis domain; H – histidine kinase domain; S – STAS domain; Pgb – protoglobin; Sgb – single domain sensor globin; U – unidentified domain. Abbreviations: ACI – Acidobacteria; Aci – Acidithiobacillales; ACTI – Actinobacteria; Actm – Actinomycete; Alt - Altermonadales; APR – Alphaproteobacteria; ARCH – Archaea; Cre – Crenarchaeota; CYA – Cyanobacteria; Eur – Euryarchaeota; BPR – Betaproteobacteria; GPR – gammaproteobacteria; Dbac – Desulfobacterales; DPR – Deltaproteobacteria; BAC – Bacteroidetes; Bur – Burkholderiales; Caul – Caulobacterales; CHLO – Chloroflexi; Chr – Chromatiales if GPR, Chrooccacales if CYA; CHL – Chlamydia/Verrumicrobia; Clo – Clostridiales; CYA – Cyanobacteria; DEI – Deinococcus; Dmon – Desulfomonadales; Ent – Enterobacteriales; EUR – Euryarchaeotes; FIR – Firmicutes; Gal – Gallionellales; Leg – Legionellales; LENT – Lentisphaerae; Metc – Methylococcales; Met – Methylophilales; Myx – Myxococcales; Nei – Neisseriales; NITR - Nitrospirae; Nos – Nostocales; Oce – Oceanospirillales; Osc – Oscillatoriales; PLA – Planctomycete; Pse – Pseudomonadales; Rhi – Rhizobiales; Rho – Rhodobacterales; Rsp – Rhodospirillales; Ric – Rickettsiales; Rub – Rubrobacterales; THE – Thermus; Thi – Thiotrichales; VER – Verrumicrobia; Vib – Vibrionales; Xan - Xanthomonadales.
(TIF)

Figure S9 **NJ phylogenetic tree of fungal and bacterial Sgbs.** NJ tree of a TCOFFEE v. 9.01 alignment of 51 fungal, 57 bacterial (including 16 Pgbs), one rotifer and one heterolobosan Sgbs, using two Adgbs [16] as outgroup. Only bootstrap values >50% are shown. The sequences are identified by the first three letters of the binary species name, the number of residues, and the first three or four letters of the phylum, followed by the first three letters of the family (see Table S1). Abbreviations: FINSE - Fungi incertae sedis; ASC – Ascomycota; Dot – Dothideomycetes; Eur – Eurotiomycetes; Pez – Pezizomycete; Sac – Saccharomycetes; Sor – Sordariomycetes; BAS – Basidiomycota; Aga – Agaromycotina; Puc – Pucciniomycotina; BLA – Blastocladiomycota; CHLO – Chloroflexi; CHY – Chytridiomycota; HETER – Heterolobosan; ONY – Onygenales; PEZ – Pezizomycotina; ROTI - Rotifer. Abbreviations: ACI – Acidobacteria; Aci – Acidithiobacillales; ACTI – Actinobacteria; Actm – Actinomycete; Alt - Altermonadales; APR – Alphaproteobacteria; ARCH – Archaea; Cre – Crenarchaeota; Eur – Euryarchaeota; BPR – Betaproteobacteria; GPR – gammaproteobacteria; Dbac – Desulfobacterales; DPR – Deltaproteobacteria; BAC – Bacteroidetes; Bur – Burkholderiales; Caul – Caulobacterales; CHLO – Chloroflexi; Chr – Chromatiales if GPR, Chrooccacales if CYA; CHL – Chlamydia/Verrumicrobia; Clo – Clostridiales; CYA – Cyanobacteria; DEI – Deinococcus; Dmon – Desulfomonadales; Ent – Enterobacteriales; EUR – Euryarchaeotes; FIR – Firmicutes; Gal – Gallionellales; Hal – Halobacteriales; Leg – Legionellales; LENT – Lentisphaerae; Metc – Methylococcales; Met – Methylophilales; Myx – Myxococcales; Nei – Neisseriales; Nit – Nitrosomonadales; NITR - Nitrospirae; Nos – Nostocales; Oce – Oceanospirillales; Osc – Oscillatoriales; PLA – Planctomycete; Pse – Pseudomonadales; Rhi – Rhizobiales; Rho – Rhodobacterales; Rsp – Rhodospirillales; Ric – Rickettsiales; Rub – Rubrobacterales; THE – Thermus; Thi – Thiotrichales; VER – Verrumicrobia; Vib – Vibrionales; Xan - Xanthomonadales.
(TIF)

Figure S10 **NJ phylogenetic tree of fungal and bacterial T1 globins.** NJ tree of a TCOFFEE v. 9.01 alignment of 2 fungal (red and black arrows), 70 bacterial (blue), 4 euryarchaeote (purple) and 10 chlorophyte (green) T1 globins, using 2 *Physcomitrella* nsHbs as outgroup. Only bootstrap values >50% are shown. The sequences are identified by the first three letters of the binary species name, the number of residues, and the first three or four letters of the phylum, followed by the first three letters of the family (see Table S1). Abbreviations: FINSE - Fungi incertae sedis; ASC – Ascomycota; Dot – Dothideomycetes; Eur – Eurotiomycetes; Pez – Pezizomycete; Sac – Saccharomycetes; Sor – Sordariomycetes; BAS – Basidiomycota; Aga – Agaromycotina; Puc – Pucciniomycotina; BLA – Blastocladiomycota; CHLO – Chloroflexi; CHY – Chytridiomycota; HETER – Heterolobosan; ONY – Onygenales; PEZ – Pezizomycotina; ROTI - Rotifer. Abbreviations: ACI – Acidobacteria; Aci – Acidithiobacillales; ACTI – Actinobacteria; Actm – Actinomycete; Alt - Altermonadales; APR – Alphaproteobacteria; ARCH – Archaea; Cre – Crenarchaeota; Eur – Euryarchaeota; BPR – Betaproteobacteria; GPR – gammaproteobacteria; Dbac – Desulfobacterales; DPR – Deltaproteobacteria; BAC – Bacteroidetes; Bur – Burkholderiales; Caul – Caulobacterales; CHLO – Chloroflexi; Chr – Chromatiales if GPR, Chrooccacales if CYA; CHL – Chlamydia/Verrumicrobia; Clo – Clostridiales; CYA – Cyanobacteria; DEI – Deinococcus; Dmon – Desulfomonadales; Ent – Enterobacteriales; EUR – Euryarchaeotes; FIR – Firmicutes; Gal – Gallionellales; Hal – Halobacteriales; Leg – Legionellales; LENT – Lentisphaerae; Metc – Methylococcales; Met – Methylophilales; Myx – Myxococcales; Nei – Neisseriales; Nit – Nitrosomonadales; NITR - Nitrospirae; Nos – Nostocales; Oce – Oceanospirillales; Osc – Oscillatoriales; PLA – Planctomycete; Pse – Pseudomonadales; Rhi – Rhizobiales; Rho – Rhodobacterales; Rsp – Rhodospirillales; Ric – Rickettsiales; Rub – Rubrobacterales; THE – Thermus; Thi – Thiotrichales; VER – Verrumicrobia; Vib – Vibrionales; Xan - Xanthomonadales.
(TIF)

Table S1 **Identified and putative globins in fungal genomes.**
(DOCX)

Table S2 **MUMSA scores for the five multiple alignment algorithms used in this work.**
(DOCX)

Table S3 **Hits obtained via PSIBLAST 2^nd^ iteration using** ***Aspergillus clavatus*** **(Ascomycota) FHb globin domain (XP_001274889.1), as query, and selecting the first 40 fungal FHbs for the 2^nd^ iteration.**
(DOCX)

Table S4 **Hits obtained via PSIBLAST 2^nd^ iteration using** ***Ajellomyces dermatidis*** **(Ascomycota; Pezizomycotina; Eurotiomycetes) Sgb, 214aa (33–214) (XP_002625170.1), as query.**
(DOCX)

Table S5 **Hits obtained via PSIBLAST 2^nd^ iteration using** ***Coprinopsis cinerea*** **(Basidiomycota) Sgb, 235aa (26–194) (XP_001838134.1), as query.**
(DOCX)

Table S6 **Hits obtained via BLASTP using the T1 globin domain from the** ***Allomyces macrogynus*** **(Blastocladiomycota) 1129aa chimeric protein (AMAG_16521T0), as query.**
(DOCX)

Table S7 **Hits obtained via BLASTP using the** ***Batrachochytrium dendrobatidis*** **(Chytridiomycota) T1 globin (BDEG_06358), as query.**
(DOCX)
