# Supplementary figures and images for: A Phylogenetic Analysis of the Globins in Fungi
Source: PLoS One. 2012 Feb 27;7(2):e31856. doi: 10.1371/journal.pone.0031856 (PMC3287990; doi:10.1371/journal.pone.0031856)

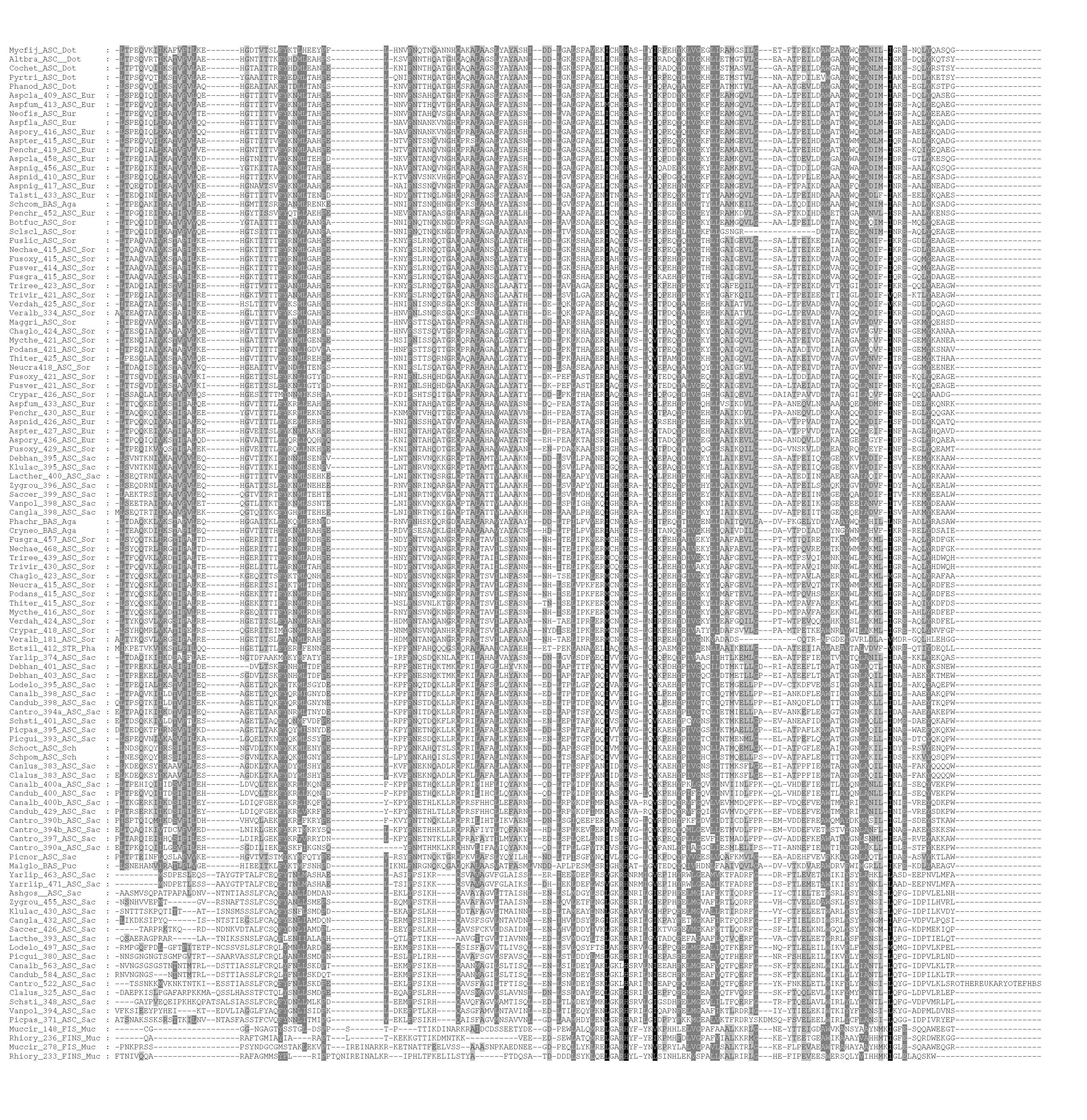

Supplement: Figure S1 — MAFFT alignments of fungal FHbs. (TIF) [file pone.0031856.s001.tif]

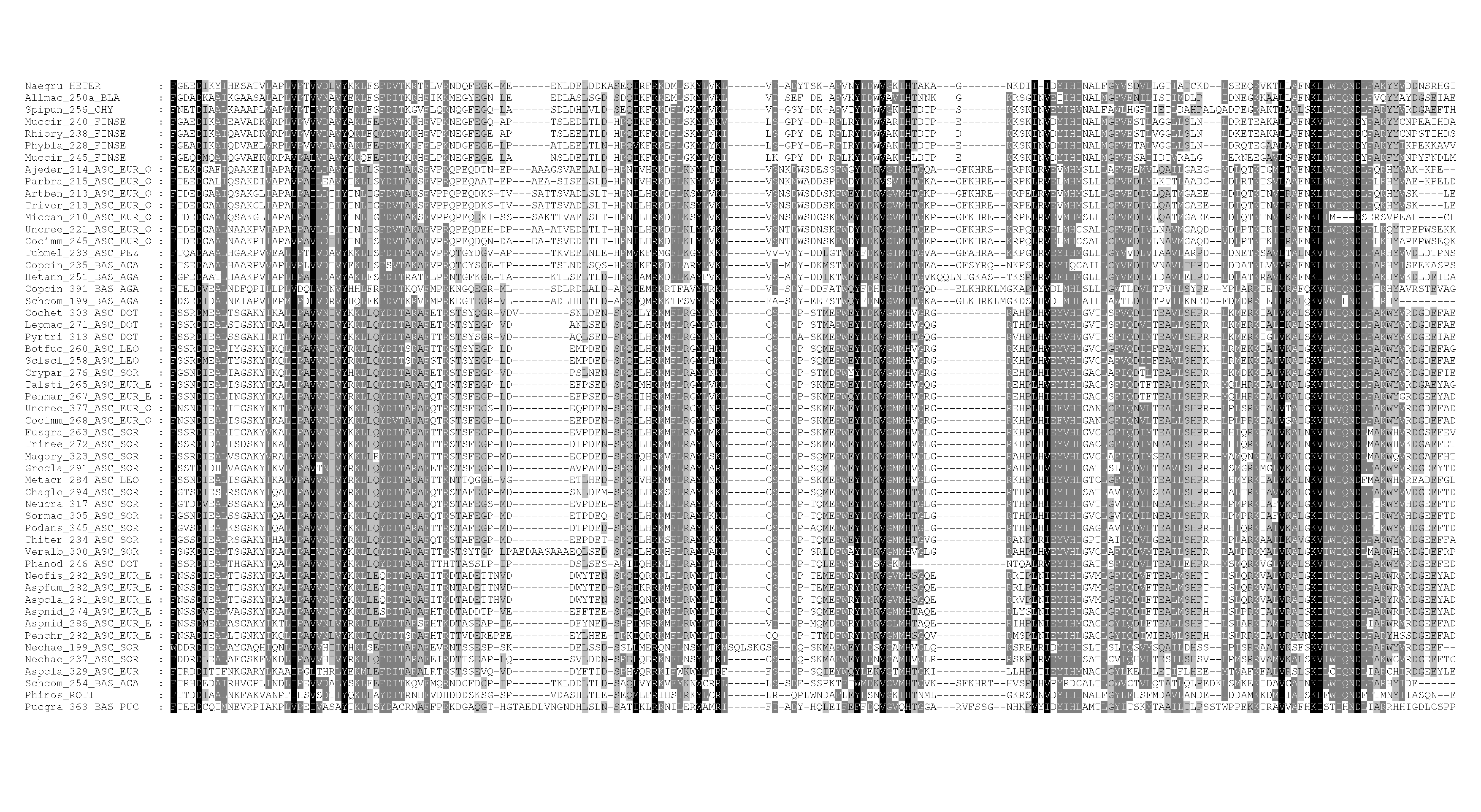

Supplement: Figure S2 — MAFFT alignments of fungal Sgbs. (TIF) [file pone.0031856.s002.tif]

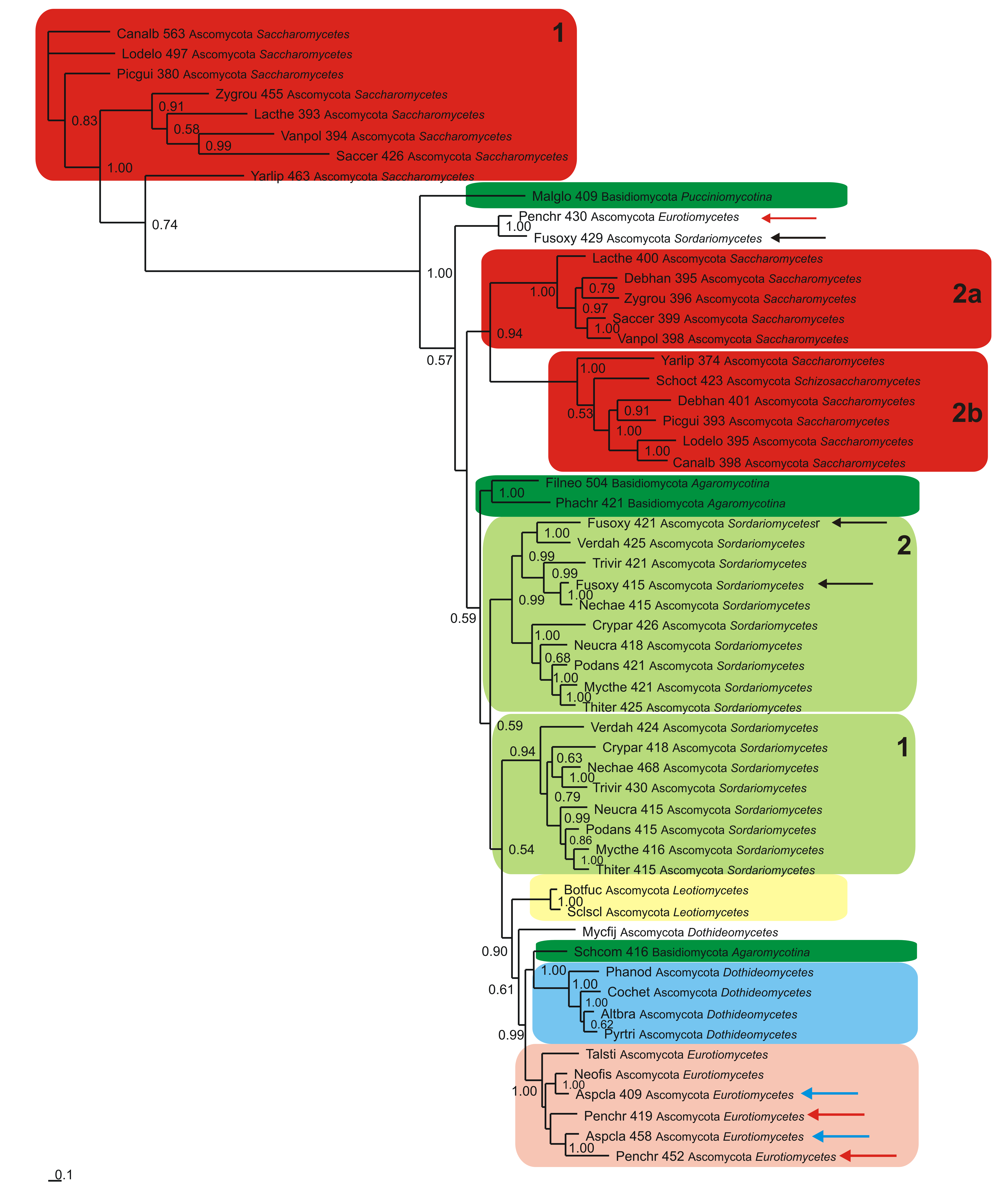

Supplement: Figure S3 — Bayesian phylogenetic tree of complete fungal FHbs. Bayesian tree of a MAFFT v.6.850 alignment of 62 complete (globin+reductase domains) fungal FHbs, including all the sequences in Fig. 3, except the Fungi incertae sedis and the two outgroup sequences. Support values at branches represent Bayesian posterior probabilities (>0.5). The sequences are identified by the first three letters of the binary species name, the number of residues, and the full phylum and family names (see Table S1). (TIF) [file pone.0031856.s003.tif]

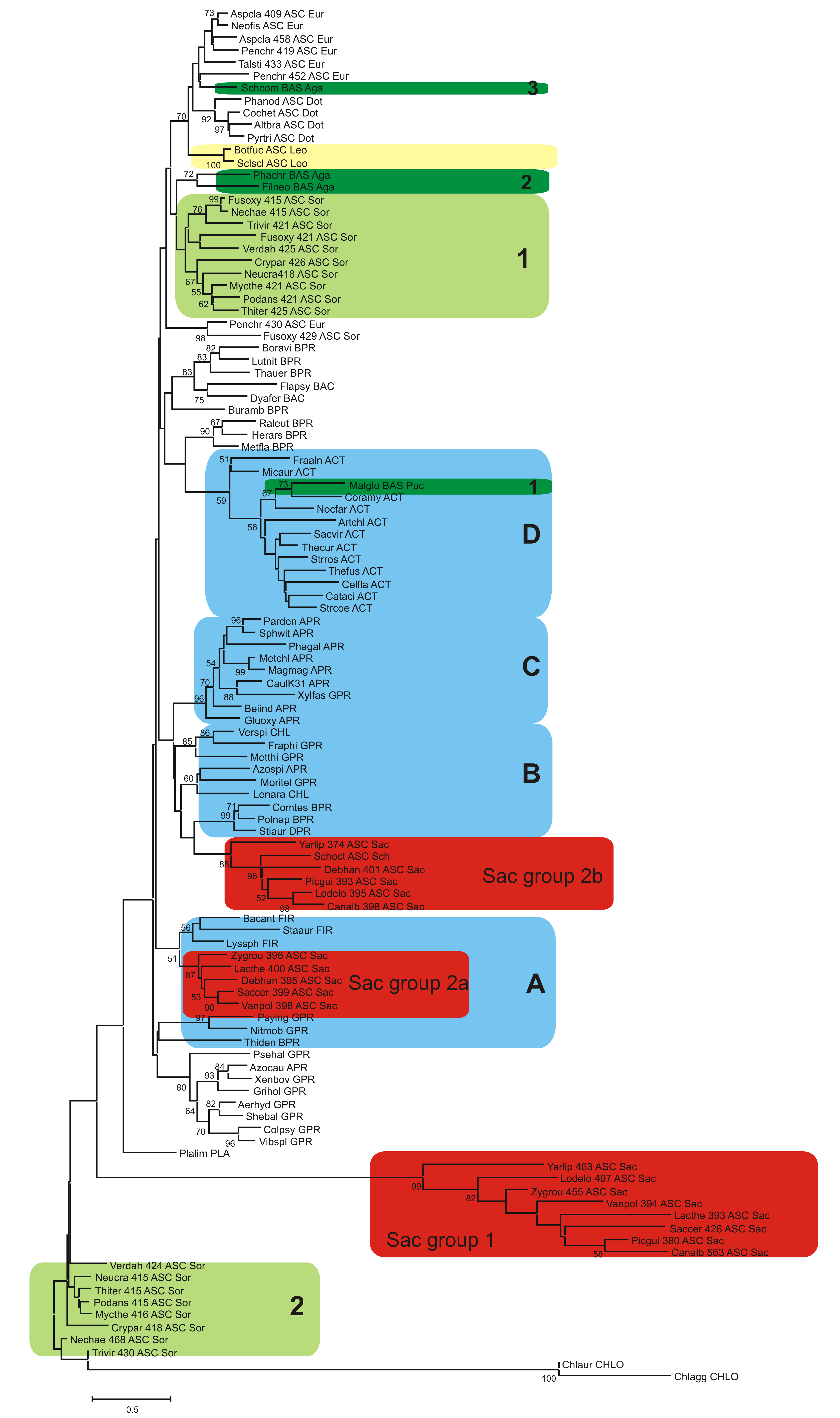

Supplement: Figure S4 — NJ phylogenetic tree of fungal and bacterial FHbs. NJ tree of a TCOFFEE v. 9.01 alignment of 37 representative fungal FHbs and 55 representative bacterial FHbs using two bacterial Pgbs as outgroup. Only bootstrap values >50% are shown. The sequences are identified by the first three letters of the binary species name, the number of residues, and the first three or four letters of the phylum, followed by the first three letters of the family (see Table S1). Abbreviations: ASC – Ascomycota; Dot – Dothideomycetes; Eur – Eurotiomycetes; Leo – Leotiomycetes; Sac – Saccharomycetes; Sor – Sordariomycetes; BAS – Basidiomycota; Aga – Agaromycotina; Puc – Pucciniomycotina; ACT – Actinobacteria; APR – Alphaproteobacteria; BPR – Betaproteobacteria; GPR – Gammaproteobacteria; DPR – Deltaproteobacteria; BAC – Bacteroidetes; CHL – Chlamydia/Verrumicrobia; FIR – Firmicutes; PLA – Planctomycete. (TIF) [file pone.0031856.s004.tif]

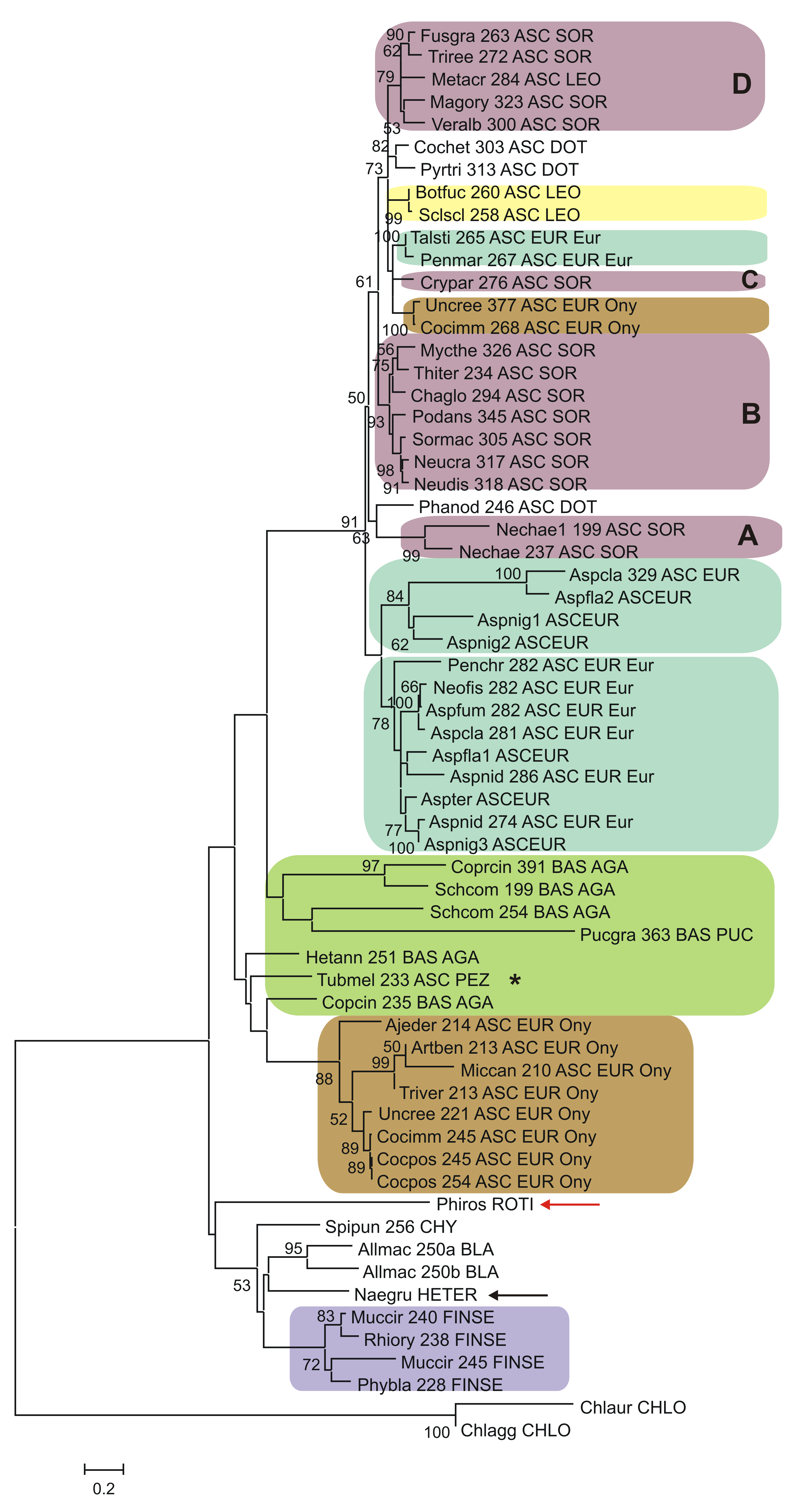

Supplement: Figure S5 — NJ phylogenetic tree of fungal Sgbs. NJ tree of a TCOFFEE v. 9.01 alignment of 59 fungal, one rotifer and one heterolobosan Sgbs. Only bootstrap values >50% are shown. The sequences are identified by the first three letters of the binary species name, the number of residues, and the first three or four letters of the phylum, followed by the first three letters of the family (see Table S1). Abbreviations: FINSE - Fungi incertae sedis; ASC – Ascomycota; Dot – Dothideomycetes; Eur – Eurotiomycetes; Pez – Pezizomycete; Sac – Saccharomycetes; Sor – Sordariomycetes; BAS – Basidiomycota; Aga – Agaromycotina; Puc – Pucciniomycotina; BLA – Blastocladiomycota; CHLO – Chloroflexi; CHY – Chytridiomycota; HETER – Heterolobosan; ONY – Onygenales; PEZ – Pezizomycotina; ROTI - Rotifer. (TIF) [file pone.0031856.s005.tif]

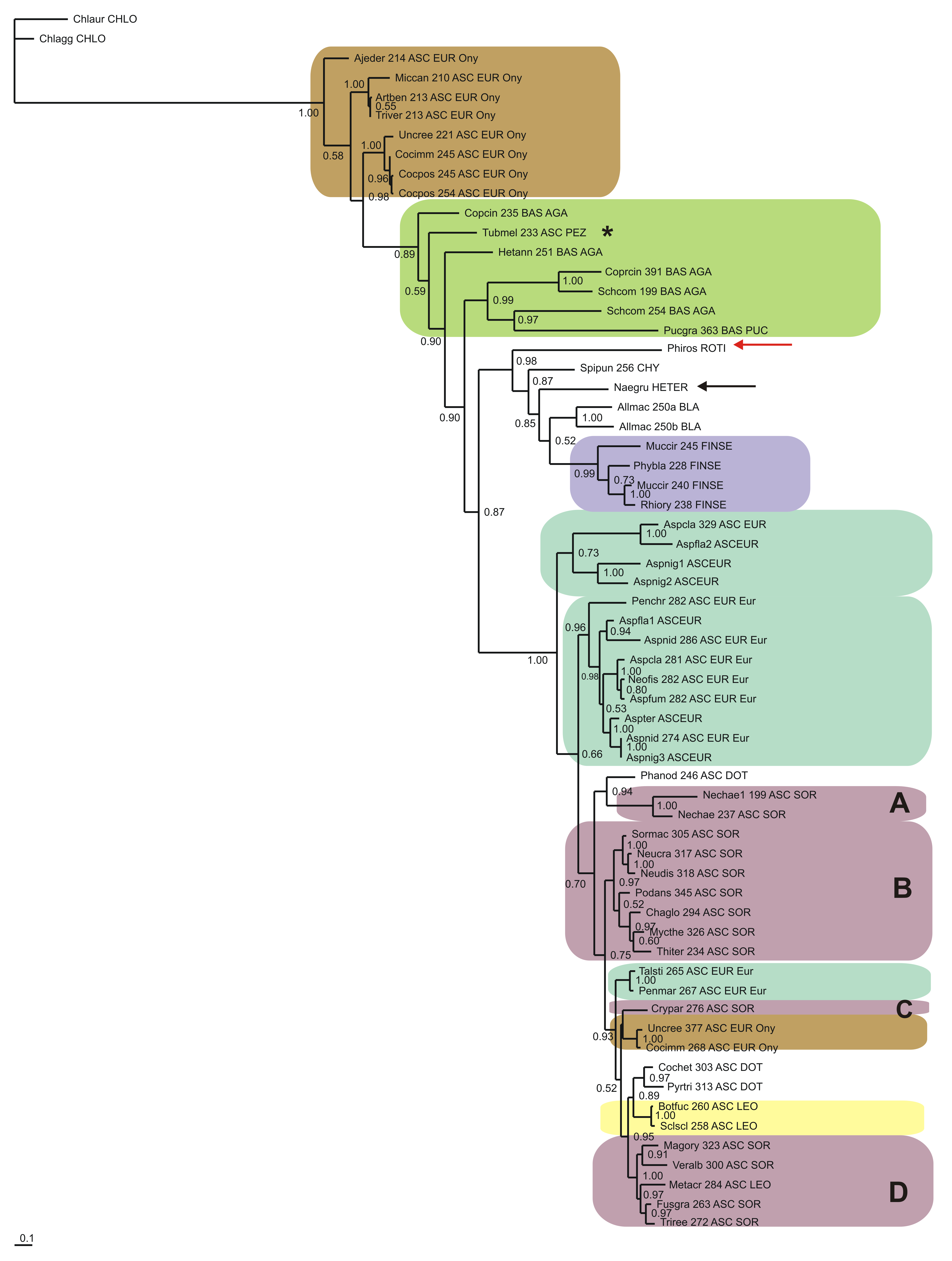

Supplement: Figure S6 — Bayesian phylogenetic tree of fungal Sgbs. Bayesian tree based on a TCOFFEE v. 9.01 alignment of 59 fungal, one rotifer and one heterolobosan Sgbs. Support values at branches represent Bayesian posterior probabilities (>0.5). The sequences are identified by the first three letters of the binary species name, the number of residues, and the full phylum and family names (see Table S1). (TIF) [file pone.0031856.s006.tif]

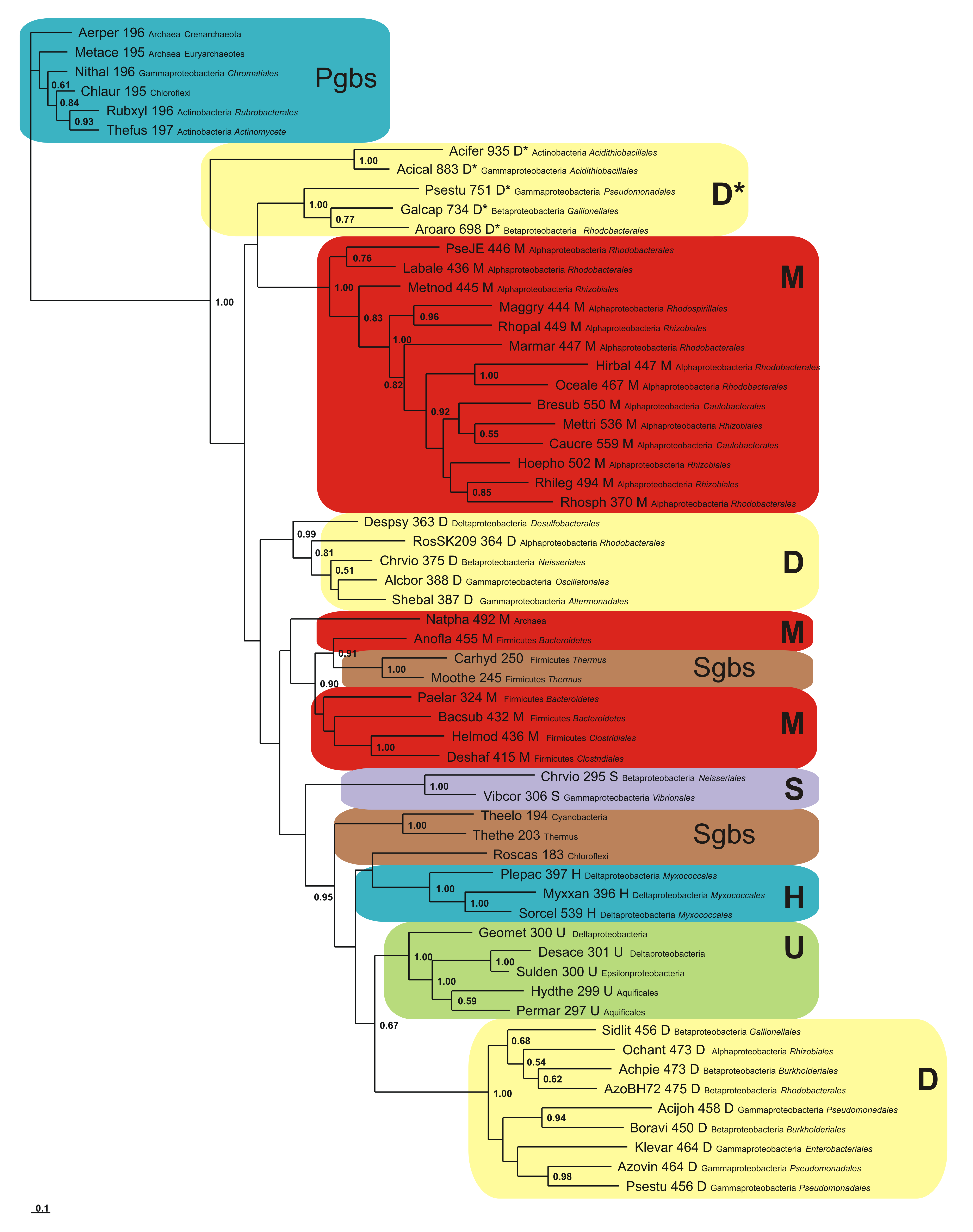

Supplement: Figure S7 — Bayesian phylogenetic tree of bacterial GCSs and Sgbs. Bayesian tree based on a MUSCLE v.6.850 alignment of 60 representative bacterial GCSs including 5 single domain sensor globins (light brown boxes). Support values at branches represent Bayesian posterior probabilities (>0.5). The first three letters of each part of the binary species name is followed by the number of residues and by a letter denoting the nonglobin domain: D – diguanylate cyclase; D* - diguanylate cyclase with additional domains; M – methyl accepting chemotaxis domain; H – histidine kinase domain; S – STAS domain; Pgb – protoglobin; Sgb – single domain sensor globin; U – unidentified domain. (TIF) [file pone.0031856.s007.tif]

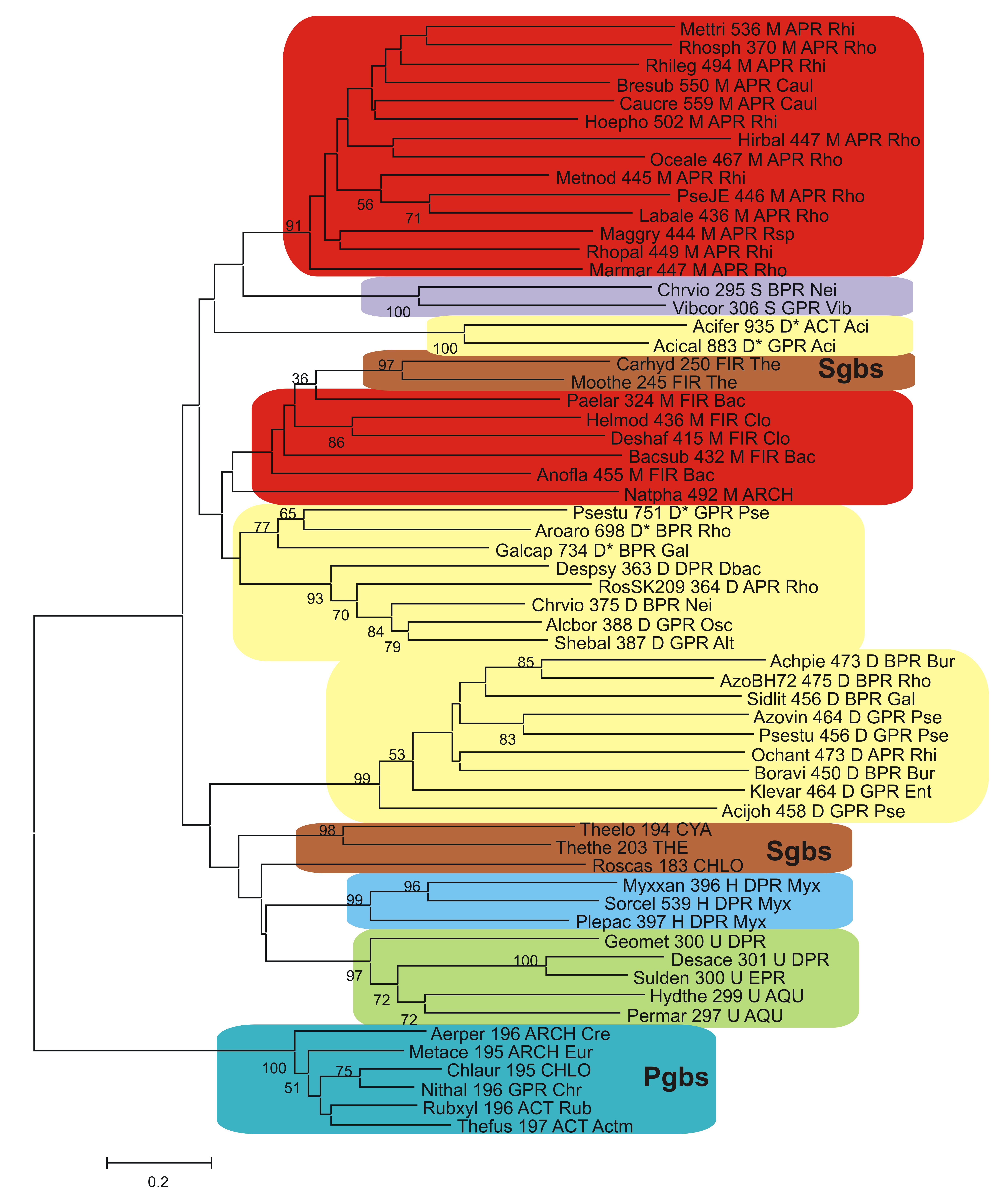

Supplement: Figure S8 — NJ phylogenetic tree of bacterial GCSs and Sgbs. NJ tree of a MUSCLE v.6.850 alignment of 60 representative bacterial GCSs including 5 single domain sensor globins (light brown boxes). Only bootstrap values >50% are shown. The first three letters of each part of the binary is followed by number of residues and by a letter denoting the nonglobin domain: D – diguanylate cyclase; D* - diguanylate cyclase with additional domains; M – methyl accepting chemotaxis domain; H – histidine kinase domain; S – STAS domain; Pgb – protoglobin; Sgb – single domain sensor globin; U – unidentified domain. Abbreviations: ACI – Acidobacteria; Aci – Acidithiobacillales; ACTI – Actinobacteria; Actm – Actinomycete; Alt - Altermonadales; APR – Alphaproteobacteria; ARCH – Archaea; Cre – Crenarchaeota; CYA – Cyanobacteria; Eur – Euryarchaeota; BPR – Betaproteobacteria; GPR – gammaproteobacteria; Dbac – Desulfobacterales; DPR – Deltaproteobacteria; BAC – Bacteroidetes; Bur – Burkholderiales; Caul – Caulobacterales; CHLO – Chloroflexi; Chr – Chromatiales if GPR, Chrooccacales if CYA; CHL – Chlamydia/Verrumicrobia; Clo – Clostridiales; CYA – Cyanobacteria; DEI – Deinococcus; Dmon – Desulfomonadales; Ent – Enterobacteriales; EUR – Euryarchaeotes; FIR – Firmicutes; Gal – Gallionellales; Leg – Legionellales; LENT – Lentisphaerae; Metc – Methylococcales; Met – Methylophilales; Myx – Myxococcales; Nei – Neisseriales; NITR - Nitrospirae; Nos – Nostocales; Oce – Oceanospirillales; Osc – Oscillatoriales; PLA – Planctomycete; Pse – Pseudomonadales; Rhi – Rhizobiales; Rho – Rhodobacterales; Rsp – Rhodospirillales; Ric – Rickettsiales; Rub – Rubrobacterales; THE – Thermus; Thi – Thiotrichales; VER – Verrumicrobia; Vib – Vibrionales; Xan - Xanthomonadales. (TIF) [file pone.0031856.s008.tif]

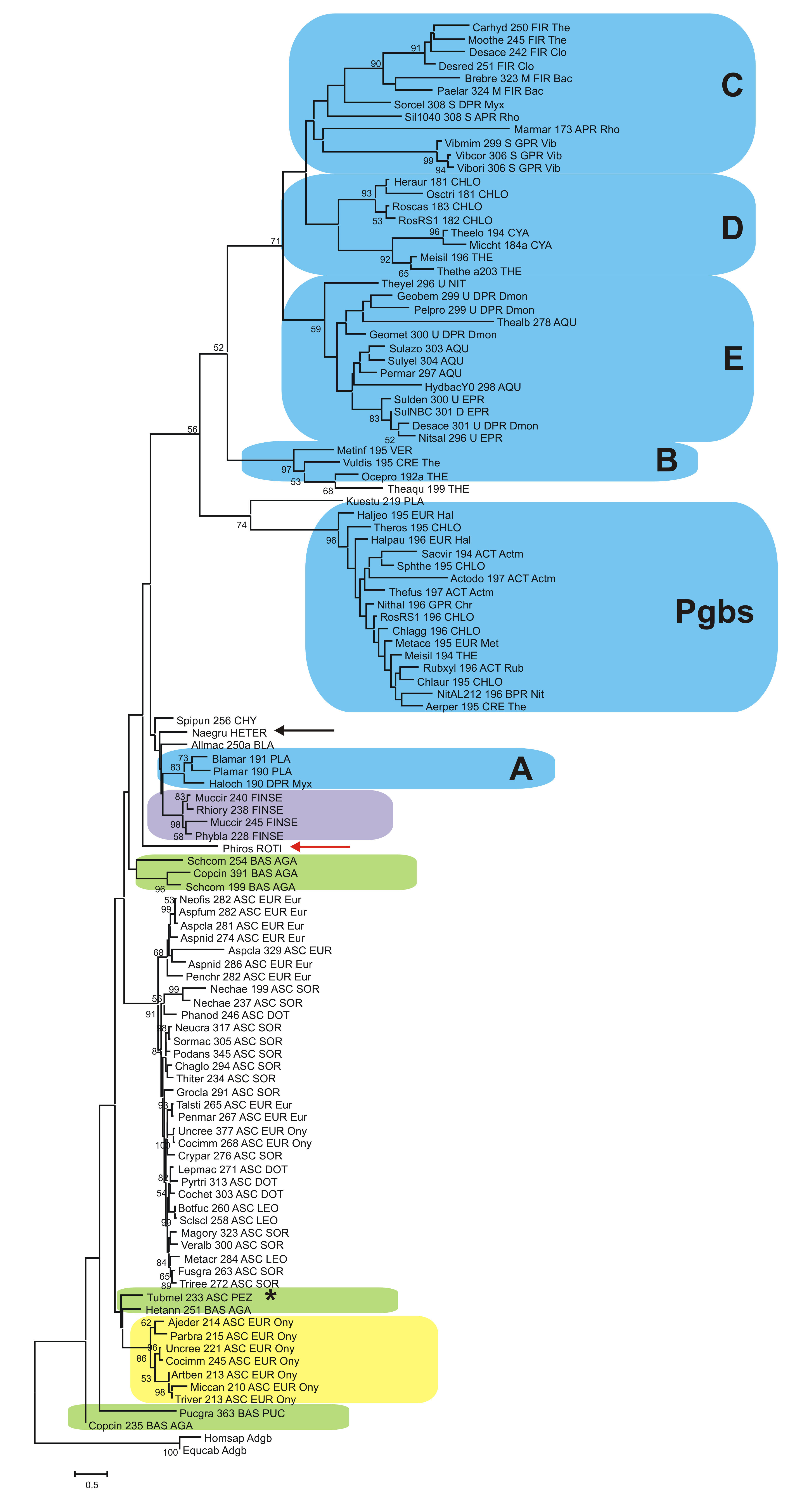

Supplement: Figure S9 — NJ phylogenetic tree of fungal and bacterial Sgbs. NJ tree of a TCOFFEE v. 9.01 alignment of 51 fungal, 57 bacterial (including 16 Pgbs), one rotifer and one heterolobosan Sgbs, using two Adgbs [16] as outgroup. Only bootstrap values >50% are shown. The sequences are identified by the first three letters of the binary species name, the number of residues, and the first three or four letters of the phylum, followed by the first three letters of the family (see Table S1). Abbreviations: FINSE - Fungi incertae sedis; ASC – Ascomycota; Dot – Dothideomycetes; Eur – Eurotiomycetes; Pez – Pezizomycete; Sac – Saccharomycetes; Sor – Sordariomycetes; BAS – Basidiomycota; Aga – Agaromycotina; Puc – Pucciniomycotina; BLA – Blastocladiomycota; CHLO – Chloroflexi; CHY – Chytridiomycota; HETER – Heterolobosan; ONY – Onygenales; PEZ – Pezizomycotina; ROTI - Rotifer. Abbreviations: ACI – Acidobacteria; Aci – Acidithiobacillales; ACTI – Actinobacteria; Actm – Actinomycete; Alt - Altermonadales; APR – Alphaproteobacteria; ARCH – Archaea; Cre – Crenarchaeota; Eur – Euryarchaeota; BPR – Betaproteobacteria; GPR – gammaproteobacteria; Dbac – Desulfobacterales; DPR – Deltaproteobacteria; BAC – Bacteroidetes; Bur – Burkholderiales; Caul – Caulobacterales; CHLO – Chloroflexi; Chr – Chromatiales if GPR, Chrooccacales if CYA; CHL – Chlamydia/Verrumicrobia; Clo – Clostridiales; CYA – Cyanobacteria; DEI – Deinococcus; Dmon – Desulfomonadales; Ent – Enterobacteriales; EUR – Euryarchaeotes; FIR – Firmicutes; Gal – Gallionellales; Hal – Halobacteriales; Leg – Legionellales; LENT – Lentisphaerae; Metc – Methylococcales; Met – Methylophilales; Myx – Myxococcales; Nei – Neisseriales; Nit – Nitrosomonadales; NITR - Nitrospirae; Nos – Nostocales; Oce – Oceanospirillales; Osc – Oscillatoriales; PLA – Planctomycete; Pse – Pseudomonadales; Rhi – Rhizobiales; Rho – Rhodobacterales; Rsp – Rhodospirillales; Ric – Rickettsiales; Rub – Rubrobacterales; THE – Thermus; Thi – Thiotrichales; VER – Verrumicrobia; Vi [file pone.0031856.s009.tif]

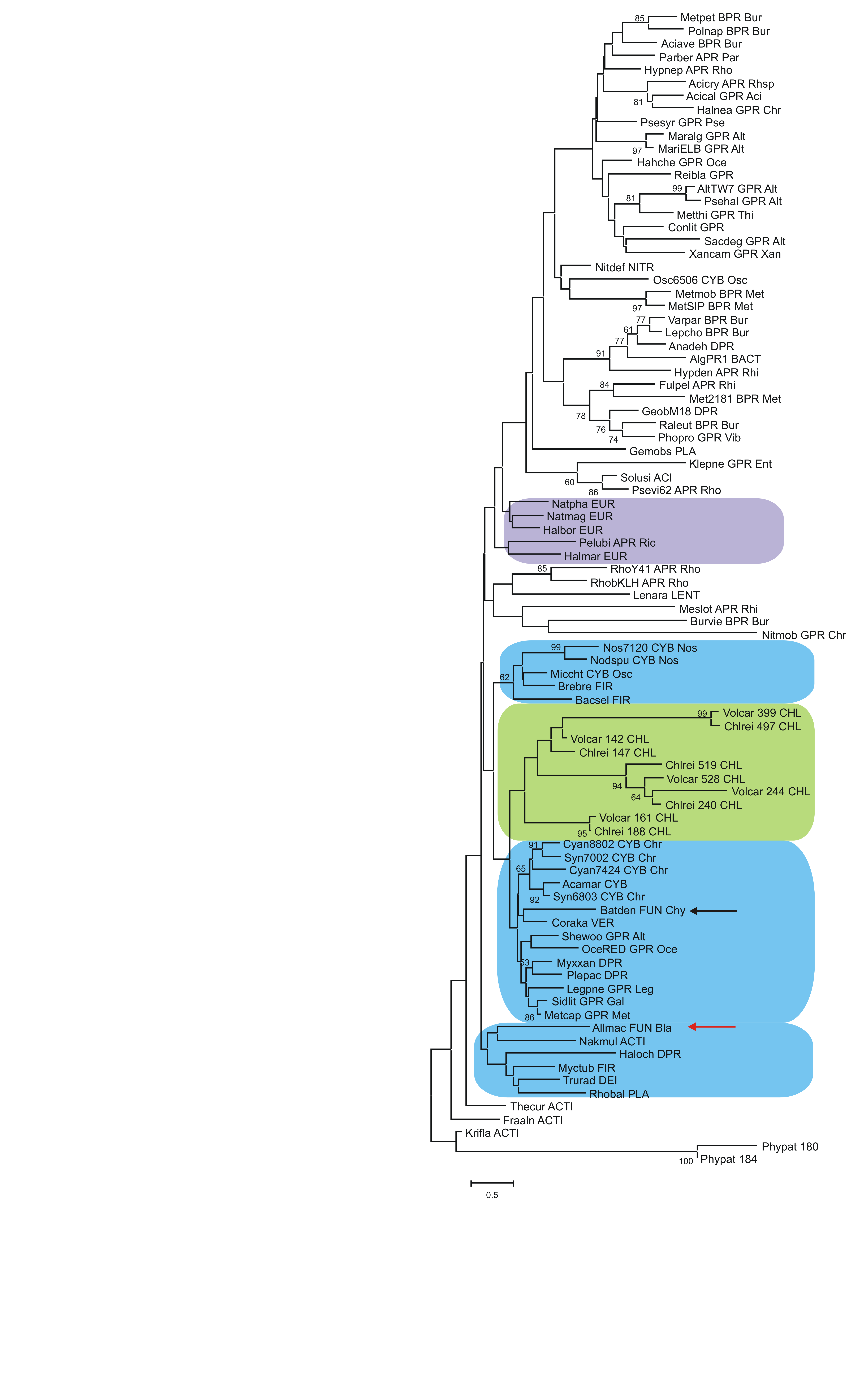

Supplement: Figure S10 — NJ phylogenetic tree of fungal and bacterial T1 globins. NJ tree of a TCOFFEE v. 9.01 alignment of 2 fungal (red and black arrows), 70 bacterial (blue), 4 euryarchaeote (purple) and 10 chlorophyte (green) T1 globins, using 2 Physcomitrella nsHbs as outgroup. Only bootstrap values >50% are shown. The sequences are identified by the first three letters of the binary species name, the number of residues, and the first three or four letters of the phylum, followed by the first three letters of the family (see Table S1). Abbreviations: FINSE - Fungi incertae sedis; ASC – Ascomycota; Dot – Dothideomycetes; Eur – Eurotiomycetes; Pez – Pezizomycete; Sac – Saccharomycetes; Sor – Sordariomycetes; BAS – Basidiomycota; Aga – Agaromycotina; Puc – Pucciniomycotina; BLA – Blastocladiomycota; CHLO – Chloroflexi; CHY – Chytridiomycota; HETER – Heterolobosan; ONY – Onygenales; PEZ – Pezizomycotina; ROTI - Rotifer. Abbreviations: ACI – Acidobacteria; Aci – Acidithiobacillales; ACTI – Actinobacteria; Actm – Actinomycete; Alt - Altermonadales; APR – Alphaproteobacteria; ARCH – Archaea; Cre – Crenarchaeota; Eur – Euryarchaeota; BPR – Betaproteobacteria; GPR – gammaproteobacteria; Dbac – Desulfobacterales; DPR – Deltaproteobacteria; BAC – Bacteroidetes; Bur – Burkholderiales; Caul – Caulobacterales; CHLO – Chloroflexi; Chr – Chromatiales if GPR, Chrooccacales if CYA; CHL – Chlamydia/Verrumicrobia; Clo – Clostridiales; CYA – Cyanobacteria; DEI – Deinococcus; Dmon – Desulfomonadales; Ent – Enterobacteriales; EUR – Euryarchaeotes; FIR – Firmicutes; Gal – Gallionellales; Hal – Halobacteriales; Leg – Legionellales; LENT – Lentisphaerae; Metc – Methylococcales; Met – Methylophilales; Myx – Myxococcales; Nei – Neisseriales; Nit – Nitrosomonadales; NITR - Nitrospirae; Nos – Nostocales; Oce – Oceanospirillales; Osc – Oscillatoriales; PLA – Planctomycete; Pse – Pseudomonadales; Rhi – Rhizobiales; Rho – Rhodobacterales; Rsp – Rhodospirillales; Ric – Rickettsiales; Rub – Rubrobacterales; THE – Thermu [file pone.0031856.s010.tif]
