# Supplementary material for: A Phylogenetic Analysis of the Globins in Fungi
Source: PLoS One. 2012 Feb 27;7(2):e31856. doi: 10.1371/journal.pone.0031856 (PMC3287990; doi:10.1371/journal.pone.0031856)
Supplement: Table S1 — Identified and putative globins in fungal genomes. (DOCX) [file pone.0031856.s011.docx]

**Table S1. Identified and putative globins in fungal genomes^1^.**

| Taxon ^2^ | **Genome**  **size, Mbp ^3^** | **Identifier** | **Size and**  **GD (aa)** | Type of globin ^4^ |
| --- | --- | --- | --- | --- |
| **Blastocladiomycota** **(BLA)**  **Blastocladiomycetes**  **Blastocladiales** |  |  |  |  |
| [*Allomyces macrogynus*](http://www.ncbi.nlm.nih.gov/portal/query.fcgi?p$site=entrez&db=genomeprj&cmd=Retrieve&dopt=Overview&list_uids=20563) | 30 C | AMAG_09320T0  AMAG_06816T0  AMAG_16521T0 | 250, 26-195  250, 26-192  1129, 826-944 | Sgb  Sgb  N-terminal ribonuclease inhibitor +T1gb |
| *Blastocladiella emersonii* | ~30 |  |  | Sgb |
| **Chytridiomycota (CHY)**  **Chytridiomycetes**  **Chytridiales** |  |  |  |  |
| *Batrachochytrium dendrobatidis* | 24.3 C | BDEG_06358 | 116 | T1gb |
| **Spizellomycetales** |  |  |  |  |
| *Spizellomyces punctatus* | 24.1 | SPPG_03693T0 | 256, 10-213 | Sgb |
| Dikarya |  |  |  |  |
| Ascomycota (ASC)  Saccharomyceta  Pezizomycotina  Leotiomyceta  Dothideomyceta  Dothideomycetes (Dot)  Dothideomycetidae |  |  |  |  |
| *Aureobasidium pullulans* | ? | ApulSEQ17250  ApulSEQ6134 |  | FHb  Sgb |
| *Cladosporium fulvum* | 61.1 | BE187718.1 |  | FHb |
| *Dothistroma septosporum* | 30.2 | jgi\|Dotse1\|70129  jgi\|Dotse1\|65494 | 415, 2-151  264, 33-199 | Complete FHb  Sgb |
| *Mycosphaerella fijiensis* | 73.4 C | jgi\|Mycfi1\|64152\| | 417, 3-149 | Complete FHb |
| *Mycosphaerella graminicola* | 39.7 C | No globins |  |  |
| *Septoria musiva* | ? | jgi\|Semu1\|132162 | 415, 2-151 | Complete FHb |
| Pleosporomycetidae |  |  |  |  |
| *Alternaria brassicicola* | 30.3 C | AB04598.1 | 419, 3-148 | Complete FHb |
| *Cochliobolus heterostrophus* | 34.9 C | [jgi\|CocheC5_1\|25727\|](http://genome.jgi-psf.org/cgi-bin/dispGeneModel?db=CocheC5_1&tid=25727)  jgi\|CocheC5_1\|25640\| | 435, 3-147  303, 35-202 | Complete FHb  SD + unknown C-terminal |
| *Leptosphaeria maculans* | 45.1 C | CBX92102.1 | 271, 34-200 | Sgb |
| *Phaeosphaeria nodorum*  *(Stagonospora nodorum)* | 36.6 C | XP_001796561.1  XP_001795267.1 | 412, 3-148  246, 31-185 | Complete FHb  Sgb |
| *Pyrenophora teres* | 42.0 | AEEY01006651.1  AEEY01000817.1 |  | FHb  Sgb |
| *Pyrenophora tritici-repentis* | 37.8 C | XP_001931846.1  XP_001934234.1 | 419, 3-149  313, 37-193 | Complete FHb  SD + unknown C-terminal |
| *Setosphaeria turcica* | ? | jgi\|Settu1\|85063  jgi\|Settu1\|86139 | 419, 4-145  317, 34-200 | Complete FHb  SD + unknown C-terminal |
| Eurotiomycetes (Eur) |  |  |  |  |
| Chaetothyriomycetidae |  |  |  |  |
| *Exophiala dermatidis* | ? | AFPA01000096.1 | 458, 56-204 | Complete FHb |
| Eurotiomycetidae  Eurotiales |  |  |  |  |
| Trichocomaceae |  |  |  |  |
| *Neosartorya fischeri* | 35 | XP_001262472.1  XP_001265294.1 | 413, 3-154  282, 15-204 | Complete FHb  Sgb |
| *Talaromyces stipitatus* | 35.6 | XP_002482681.1 XP_002483906.1 | 433, 19-174  265, 65-231 | Complete FHb  Sgb |
| Mitosporic Trichocomaceae |  |  |  |  |
| *Aspergillus clavatus* | 35 C | XP_001274889.1  XP_001268526.1  XP_001269793.1  XP_001276705.1 | 409, 3-148  458, 45-192  281, 15-204  329, 2-192 | Complete FHb  FHb with N-terminal signal sequence  Sgb  SD + unknown C-terminal |
| *Aspergillus flavus* | 36 C | XP_002377474.1  XP_002375473.1 XP_002374341.1 XP_002375155.1 | 416, 1-146  436, 34-179  285, 37-195  373, 36-202 | Complete FHb  FHb with N-terminal signal sequence  Sgb  SD + unknown C-terminal |
| *Aspergillus fumigatus* | 29.4 C | XP_746528.1  XP_747310.1  XP_750111.1 | 413, 3-154  433, 31-184  282, 10-204 | Complete FHb  FHb with N-terminal signal sequence  Sgb |
| *Aspergillus nidulans* | 30.2 C | XP_664773.1  XP_661126.1  XP_001398867.1 XP_661655.1 | 410, 2-145  426, 25-166  274, 37-194  286, 17-208 | Complete FHb  FHb with N-terminal signal sequence  Sgb  Sgb |
| *Aspergillus niger* | 34.0 C | XP_001400872.1  XP_001398637.2  XP_001398867.1  XP_001393356.1  XP_001389852.1  XP_001396525.1 | 417, 2-146  439, 48-192  274, 3-194  302, 6-194  313, 3-195  417, 3-194 | Complete FHb  FHb with N-terminal signal sequence  Sgb  SD + unknown C-terminal  SD + unknown C-terminal  SD + unknown C-terminal |
| *Aspergillus oryzae* | 37.0 C | XP_001825874.1 XP_001727230.1  XP_001820095.1 | 416. 3-154  436, 34-181  279, 2-195 | Complete FHb  FHb with N-terminal signal sequence  Sgb |
| *Aspergillus terreus* | 29.3 C | XP_001211325.1  XP_001216520.1  XP_001213026.1 | 416, 3-154  427, 27-178  275, 2-194 | Complete FHb  FHb with N-terminal signal sequence  Sgb |
| *Penicillium chrysogenum* | 32.2 C | XP_002558266.1 XP_002559620.1  XP_002561314.1  XP_002562107.1 | 419, 6-161  430, 26-172  452, 52-199  282, 36-203 | Complete FHb  FHb with N-terminal signal sequence  FHb with N-terminal signal sequence  Sgb |
| *Penicillium marneffei* | 28.6 | XP_002148409.1 XP_002150353.1 | 416, 2-147  267, 67-233 | Complete FHb  Sgb |
| Onygenales (Ony) |  |  |  |  |
| *Ajellomyces dermatitidis* | 67-75 | XP_002625170.1 | 214, 33-214 | Sgb |
| *Arthroderma benhamie* | 23 | XP_003016268.1 | 213, 33-213 | Sgb |
| *Arthroderma gypseum* | 23 C | XP_003176947.1 | 212, 33-213 | Sgb |
| *Arthroderma otae* | 23 | XP_002850838.1 | 210, 33-210 | Sgb |
| *Ascosphaera apis* | 24 | AARE01001462.1 |  | Sgb |
| *Microsporum canis* | 23.3 | EEQ28054.1 | 210, 33-210 | Sgb, 16aa insert between helices BC-E |
| *Trichophyton equinum* | 24.2 C | ABWI01000223.1 |  | Sgb |
| *Trichophyton rubrum* | 22 | ACPH01000027.1 |  | Sgb |
| *Trichophyton tonsurans* | 22 | ACPI01000623.1 |  | Sgb |
| *Trichophyton verrucosum* | 22 | XP_003024623.1 | 213, 33-213 | Sgb |
| *Uncinocarpus reesii* | 22.3 C | XP_002542547.1 XP_002543129.1 | 221, 33-213  377, 35-193 | Sgb  SD + unknown C-terminal |
| **Mitosporic Onygenales** |  |  |  |  |
| *Coccidioides immitis* | 27.6 – 28.8 C | XP_001244432.1  XP_001241490.1 | 245, 2-238  268, 2-193 | Sgb  Sgb |
| *Coccidioides posadasii* | 26.2 – 28.7 C | XP_001244432.1  XP_001241490.1 | 245, 56-235  268, 25-192 | Sgb  Sgb |
| *Paracoccidioides brasiliensis* | 29.1- 33.0 | EEH19835.1 | 215, 35-215 | Sgb |
| **Sordariomyceta**  **Leotiomycetes (Leo)** |  |  |  |  |
| **Erysiphales** |  |  |  |  |
| *Blumeria graminis* | ~45 | No globins |  |  |
| *Erysiphe pisi* | ? | No globins |  |  |
| **Helotiales** |  |  |  |  |
| *Botryotinia fuckeliana*  *(Botrytis cinerea)* | 38 C | XP_001557794.1  CAP74387.1  XP_001548802.1 | 328, 1-150  412, 2-147  260, 31-189 | FHb (deletions in glb and central domains  Complete FHb  Sgb |
| *Sclerotinia sclerotiorum* | 38.3 | XP_001588433.1  XP_001591454.1 | 401, 2-137  258, 31-189 | Complete FHb  Sgb |
| **Leotiomycete incertae sedis** |  |  |  |  |
| *Amorphotheca resinae* | ? | AresSEQ9933 |  | FHb |
| *Geomyces destructans* | ? | AEFC01001622.1 | 440, 42-188 | Complete FHb |
| *Geomyces pannorum* | ? | GpanSEQ7323 |  | FHb |
| **Sordariomycetes (Sor)**  **Hypocreomycetidae** |  |  |  |  |
| **Hypocreales** |  |  |  |  |
| *Aciculosporium take* | ? | AFQZ01000080.1 | 463, 28-173 | Complete FHb |
| *Cordyceps militaris* | ? | EGX90963.1 | 427, 2-146 | Complete FHb |
| *Epichloe festucae* | 26.5 C | ADFL01000904.1 | 480, 57-201 | Complete FHb |
| *Fusarium graminearum*  *(Gibberella zeae)* | 36.3 C | XP_384634.1  XP_380941.1  XP_388101.1 | 415, 3-151  457, 3-149  263, 1-189 | Complete FHb  Complete FHb  Sgb |
| *Giberella moniliformis*  *(Fusarium verticillioides)* | 41.9 | [FVEG_11186](http://www.broad.mit.edu/annotation/genome/fusarium_group/FeatureSearch.html?formids=If%2CHidden%2CIf_0%2CIf_1%2CIf_2%2CIf_3%2CIf_4%2CIf_5%2CIf_6%2CPropertySelection%2CIf_7%2CIf_8%2CIf_9%2CIf_10%2CTextField%2CIf_11%2CIf_12%2CIf_13%2CIf_14%2CIf_15%2CIf_16%2CIf_17%2CTextField_0%2CIf_18%2CIf_19%2CTextField_1%2CIf_20%2CIf_21%2CIf_22%2CTextField_2%2CIf_23%2CIf_24%2CIf_25%2CTextField_3%2CIf_26%2CIf_27%2CIf_28%2CTextField_4%2CIf_29%2CIf_30%2CTextField_5%2CTextField_6%2CSubmit&component=searchForm.%24Form&service=direct&session=T&reservedids=dbAlias%2Cdomain%2ClocalName%2CobjectId&submitmode=submit&submitname=&If=F&Hidden=*5350_252&If_0=T&If_1=T&If_2=F&If_3=F&If_4=F&If_5=T&If_6=T&If_7=F&If_8=F&If_9=F&If_10=T&If_11=F&If_12=F&If_13=F&If_14=T&If_15=T&If_16=T&If_17=T&If_18=T&If_19=T&If_20=T&If_21=T&If_22=T&If_23=T&If_24=T&If_25=T&If_26=T&If_27=T&If_28=T&If_29=T&If_30=T&PropertySelection=Gene&TextField_0=&TextField_1=&TextField_2=&TextField_3=&TextField_4=&TextField_5=0&TextField_6=0&Submit=Submit&TextField=FVEG_11186)  [FVEG_13827](http://www.broad.mit.edu/annotation/genome/fusarium_group/FeatureSearch.html?formids=If%2CHidden%2CIf_0%2CIf_1%2CIf_2%2CIf_3%2CIf_4%2CIf_5%2CIf_6%2CPropertySelection%2CIf_7%2CIf_8%2CIf_9%2CIf_10%2CTextField%2CIf_11%2CIf_12%2CIf_13%2CIf_14%2CIf_15%2CIf_16%2CIf_17%2CTextField_0%2CIf_18%2CIf_19%2CTextField_1%2CIf_20%2CIf_21%2CIf_22%2CTextField_2%2CIf_23%2CIf_24%2CIf_25%2CTextField_3%2CIf_26%2CIf_27%2CIf_28%2CTextField_4%2CIf_29%2CIf_30%2CTextField_5%2CTextField_6%2CSubmit&component=searchForm.%24Form&service=direct&session=T&reservedids=dbAlias%2Cdomain%2ClocalName%2CobjectId&submitmode=submit&submitname=&If=F&Hidden=*5350_252&If_0=T&If_1=T&If_2=F&If_3=F&If_4=F&If_5=T&If_6=T&If_7=F&If_8=F&If_9=F&If_10=T&If_11=F&If_12=F&If_13=F&If_14=T&If_15=T&If_16=T&If_17=T&If_18=T&If_19=T&If_20=T&If_21=T&If_22=T&If_23=T&If_24=T&If_25=T&If_26=T&If_27=T&If_28=T&If_29=T&If_30=T&PropertySelection=Gene&TextField_0=&TextField_1=&TextField_2=&TextField_3=&TextField_4=&TextField_5=0&TextField_6=0&Submit=Submit&TextField=FVEG_13827) | 414, 3-148  421, 3-148 | Complete FHb  Complete FHb |
| *Metarhizium acridum* | ? | EFY91059  EFY88256.1 | 415, 1-134  284, 32-198 | Complete FHb  Sgb |
| *Metarhizium anisopliae (15)* | 21.8 – 38.9 | EFZ04256.1  EFY95878.1 | 360, 75-205  299, 53-219 | FHb with incomplete C-terminal  Sgb |
| *Nectria haematococca* | 54.4 C | EEU42675.1 EEU48840.1  XP_003042020.1  XP_003047909.1 | 415, 3-143  468, 3-148  199, 26-198  237, 30-196 | Complete FHb  Complete FHb  Sgb  Sgb |
| *Trichoderma atroviride* | 36.1 | jgi\|Triat1\|154128\|  jgi\|Triat1\|137358\| | 421, 1-148  449, 2-148 | Complete FHb  Complete FHb |
| *Trichoderma reesei*  *(Hypocrea jecorina)* | 33.0 C | jgi\|Trire2\|123251\|  jgi\|Trire2\|76722\|  jgi\|Trire2\|82032\| | 423, 4-150  439, 3-150  272, 1-191 | Complete FHb  Complete FHb  Sgb |
| *Trichoderma virens*  *(Hypocrea virens)* | 35.0 C | Jgi\|Trive1\|80534\|  jgi\|Trive1\|83817 | 421, 3-149  430, 2-147 | Complete FHb  Complete FHb |
| **Mitosporic Hypocreales** |  |  |  |  |
| *Fusarium lichenicola* | ? | ACP18865.1 | 416, 3-148 | Complete FHb |
| *Fusarium oxysporum* | 61.4 C | BAA33011  [FOXG_15840](http://www.broad.mit.edu/annotation/genome/fusarium_group/FeatureSearch.html?formids=If%2CHidden%2CIf_0%2CIf_1%2CIf_2%2CIf_3%2CIf_4%2CIf_5%2CIf_6%2CPropertySelection%2CIf_7%2CIf_8%2CIf_9%2CIf_10%2CTextField%2CIf_11%2CIf_12%2CIf_13%2CIf_14%2CIf_15%2CIf_16%2CIf_17%2CTextField_0%2CIf_18%2CIf_19%2CTextField_1%2CIf_20%2CIf_21%2CIf_22%2CTextField_2%2CIf_23%2CIf_24%2CIf_25%2CTextField_3%2CIf_26%2CIf_27%2CIf_28%2CTextField_4%2CIf_29%2CIf_30%2CTextField_5%2CTextField_6%2CSubmit&component=searchForm.%24Form&service=direct&session=T&reservedids=dbAlias%2Cdomain%2ClocalName%2CobjectId&submitmode=submit&submitname=&If=F&Hidden=*5350_252&If_0=T&If_1=T&If_2=F&If_3=F&If_4=F&If_5=T&If_6=T&If_7=F&If_8=F&If_9=F&If_10=T&If_11=F&If_12=F&If_13=F&If_14=T&If_15=T&If_16=T&If_17=T&If_18=T&If_19=T&If_20=T&If_21=T&If_22=T&If_23=T&If_24=T&If_25=T&If_26=T&If_27=T&If_28=T&If_29=T&If_30=T&PropertySelection=Gene&TextField_0=&TextField_1=&TextField_2=&TextField_3=&TextField_4=&TextField_5=0&TextField_6=0&Submit=Submit&TextField=FOXG_15840) | 415, 3-148  429, 28-175 | Complete FHb  FHb with N-terminal signal sequence |
| **Hypocreomycetidae Incertae sedis** |  |  |  |  |
| *Glomerella cingulata* | ? | ET594190.1 |  | FHb |
| *Glomerella graminicola* | 57 | EFQ33638.1 | 420, 3-148 | Complete FHb |
| **Sordariomycetidae**  **Diaporthales** |  |  |  |  |
| *Cryphonectria* *parasitica* | 43.9 | jgi\|Crypa1\|33217\|  jgi\|Crypa1\|38042\|  jgi\|Crypa1\|48659\| | 418, 4-151  426, 3-153  276, 27-186 | Complete FHb  Complete FHb  Sgb |
| *Ophiognomonia clavigignenti-*  *juglandacearum* | ? | AEGN01016025.1 | 142 | Fgb missing helices A and B |
| **Magnaporthales** |  |  |  |  |
| *Magnaporthe oryzae*  *(Magnaporthe grisea)* | 37.8 C | XP_369046.1  XP_360903.1 | 447, 28-180  323, 1-190 | FHb with N-terminal signal sequence  SD + unknown C-terminal |
| *Magnaporthe poae* | ? | ADBL01001493.1  ADBL01000873.1 | 382, 1-115 | FHb missing helices A and B  Sgb |
| *Gaeumannomyces graminis* | 40 | ADBI01000222.1 | 523, 100-246 | Complete FHb |
| **Ophiostomatales** |  |  |  |  |
| *Grosmannia clavigera* | 32 | ACYC01000128.1  EFX02346.1 | 291, 33-200 | FHb  Sgb |
| *Ophiostoma piliferum* | ? | OpilSEQ5612 |  | Sgb |
| **Sordariales**  **Chaetomiaceae** |  |  |  |  |
| *Chaetomium globosum* | 36 | XP_001220963.1  XP_001228021.1  XP_001222087.1 | 423, 2-150  424, 2-148  294, 1-182 | Complete FHb  Complete FHb  SD + unknown C-terminal |
| *Myceliophthora* *thermophila*  *(Sporotrichum thermophila,*  *Thielavia heterothallica)* | 38.7 | jgi\|Spoth1\|61871\|  jgi\|Spoth1\|112322\|  jgi\|Spoth1\|110640\| | 416, 2-146  421, 3-147  326, 7-200 | Complete FHb  Complete FHb  SD + unknown C-terminal |
| *Thielavia terrestris* | ? | jgi\|Thite1\|128077\|  jgi\|Thite1\|157260\|  jgi\|Thite1\|31595\| | 415, 2-146  425, 4-150  234, 1-192 | Complete FHb  Complete FHb  Sgb |
| **Sordariacea** |  |  |  |  |
| *Neurospora crassa* | 37.1 C | XP_963988.2  XP_957939.1  XP_329286.1  XP_958332.1 | 415, 3-148  418, 3-148  537, 2-150  317, 4-199 | Complete FHb  Complete FHb  FHb identical to XP_963988.2  SD + unknown C-terminal |
| *Neurospora discreta* | 43 | jgi\|Neudi1\|162627\|  jgi\|Neudi1\|21031\|  jgi\|Neudi1\|125383\| | 418, 2-148  580, 2-146  318, 41-198 | Complete FHb  FHb + unknown C-terminal  Sgb |
| *Podospora anserina* | 35.5 C | XP_001912543.1  XP_001904160.1  XP_001907267.1 | 415, 2-147  421, 3-148  345, 33-200 | Complete FHb  Complete FHb  SD + unknown C-terminal |
| *Sordaria macrospora* | 38.7 C | CBI51078.1  CBI55368.1  CBI52107.1 | 415, 2-148  419, 2-147  305, 41-217 | Complete FHb  Complete FHb  Sgb |
| **Sordariomycetes incertae sedis** |  |  |  |  |
| *Verticillium albo-atrum* | 32.9 | EEY23787.1 EEY22838.1  XP_003000743.1 | 181, 2-142  334, 2-146  300, 32-207 | Fgb  FHb with incomplete C-terminal  SD + unknown C-terminal |
| *Verticillium dahlia* | 33.9 | VDAG06183  VDAG10220 | 424, 2-146  425, 2-146 | Complete FHb  Complete FHb |
| **Orbiliomycetes (Orb)** |  |  |  |  |
| *Arthrobotrys oligospora* | ? | EGX52249.1  EGX47796.1 | 456, 11-155  351, 43-209 | Complete FHb  Sgb |
| **Pezizomycetes (Pez)** |  |  |  |  |
| **Pezizales**  **Tuberaceae** |  |  |  |  |
| *Tuber borchii (white truffle)* | 34 | No globins? |  |  |
| *Tuber melanosporum*  *(Perigord truffle)* | 123.6 C | XP_002838864.1 | 233, 25-201 | Sgb |
| Saccharomycotina (Sac) **Saccharomycetes**  **Saccharomycetales** |  |  |  |  |
| Debaryomycetaceae |  |  |  |  |
| *Debaryomyces hansenii* | 12.2 C | XP_452175.1  XP_462633.1  XP_462620.1 | 395, 1-146  401, 14-151  403, 14-149 | Complete FHb  Complete FHb  FHb, identical to XP_462633.1 |
| *Lodderomyces elongisporus* | 15.5 | XP_001523563.1  XP_001527010.1 | 395, 11-155  497, 229-385 | Complete FHb  Unknown N-terminal + incomplete FHb |
| *Meyerozyma guilliermondii*  *(Pichia guilliermondii)*  *(Candida guilliermondii)* | 10.6 | XP_001481926.1 XP_001486347.1  XP_001482036.1  XP_001482259.1 | 218, 13-158  380, 151-283  393, 14-160  731, 9-149 | Fgb  Unknown N-terminal + incomplete FHb  Complete FHb  FHb + unknown C-terminal |
| *Scheffersomyces (Pichia) stipitis* | 15.4 C | XP_001385495.2  XP_001383940.2 | 348, 118-268  401, 14-161 | Unknown N-terminal + incomplete FHb  Complete FHb |
| *Spathaspora passalidarum* | ? | EGW34978.1  EGW34982.1  EGW33857.1 | 274, 56-203  338, 96-244  394, 9-154 | Fgb  FHb missing domain 1cqx3  Complete FHb |
| Dipodascaceae |  |  |  |  |
| *Yarrowia lipolytica* | 20.5 C | XP_502088.1  XP_502881.1  XP_499869.1 | 374, 1-138  463, 186-325  471, 194-333 | Complete FHb  Unknown N-terminal + incomplete FHb  Unknown N-terminal + incomplete FHb |
| Metschnikowiaceae |  |  |  |  |
| *Clavispora (Candida) lusitaniae* | 12.1 C | XP_002616766.1 XP_002619769.1 | 383, 12-158  325, 110-256 | Complete FHb  Unknown N-terminal + incomplete FHb |
| Saccharomycetaceae |  |  |  |  |
| *Ashbya (Eremothycium) gossypii* | 9.2 C | NP_982746.1 | 436, 184-339 | Unknown N-terminal + incomplete FHb |
| *Dekkera bruxellensis* | ? | EI016389.1 |  | FHb |
| *Kluyveromyces lactis* | 10.7 C | XP_452175.1  XP_453939.1 | 395, 1-134  430, 181-323 | Complete FHb  Unknown N-terminal + incomplete FHb |
| *Kluyveromyces waltii* | 11.0 C | Kwal_9681  Kwal_22190  Kwal_4395  Kwal_24852 | 396, 2-146  421, 172-314  460, 211-352  543, 173-315 | Complete FHb  Unknown N-terminal + incomplete FHb  Unknown N-terminal + incomplete FHb  Unknown N-terminal + incomplete FHb |
| *Lachancea thermotolerans*  *(Kluyveromyces thermotolerans)*  *(Zygosaccharomyces*  *thermotolerans)* | 10.4 C | XP_002555051.1 XP_002554200.1  XP_002552156.1 XP_002555892.1 | 393, 162-306  400, 1-147  431, 180-327  552, 170-314 | Unknown N-terminal + incomplete FHb  Complete FHb  Unknown N-terminal + incomplete FHb  Unknown N-terminal + complete FHb |
| *Pichia norvegensis* | ? | CAA48729.2 | 390, 1-144 | Complete FHb |
| *Pichia pastoris* | 9.3 C | XP_002494001.1 XP_002490302.1 | 371, 126-326  395, 1-146 | Unknown N-terminal + incomplete FHb Complete FHb |
| *Saccharomyces bayanus* | 11.5 – 11.8 C | ORFP:9664  ORFP:20532 | 399, 1-138  424, 171-303 | Complete FHb  Unknown N-terminal + incomplete FHb |
| *Saccharomyces castellii* | 11.35 C | AACF01000137.1 |  | FHb |
| *Saccharomyces cerevisiae* | 11.7 - 12.2 C | NP_011750.1  NP_014165.1 | 399, 1-148  426, 154-302 | Complete FHb  Unknown N-terminal + incomplete FHb |
| *Saccharomyces kudriavzevii* | 11.2 | AACI02000087.1 |  | FHb |
| *Saccharomyces mikatae* | 10.8 - 11.5 C | ORFP:9249  ORFP:18051 | 399, 1-138  410, 135-290 | Complete FHb  Unknown N-terminal + incomplete FHb |
| *Saccharomyces paradoxus* | 11.9 C | ORFP:9231  ORFP:18484 | 399, 1-138  426, 155-306 | Complete FHb  Unknown N-terminal + incomplete FHb |
| *Saccharomyces pastorianus* | ? | ABPO01000599.1 |  | FHb |
| **Wickerhamomycetaceae** |  |  |  |  |
| *Wickerhamomyces anomalus* | ? | AEGI01000001.1 | 417, 4-151 | Complete FHb |
| Mitosporic Saccharomycetales |  |  |  |  |
| *Candida albicans* | 14.3 C | XP_711060.1  XP_719397.1  XP_711954.1 | 398, 10-146  400, 17-155  563, 298-437 | Complete FHb  Complete FHb  Unknown N-terminal + incomplete FHb |
| *Candida dubliniensis* | 14.5 C | XP_002422285.1 XP_002422287.1  XP_002422512.1  XP_002417032.1 | 398, 9-155  400, 17-163  429, 46-195  584, 298-441 | Complete FHb  Complete FHb  FHb with N-terminal signal sequence  Unknown N-terminal + incomplete FHb |
| *Candida glabrata* | 12.3 C | XP_449066.1  XP_448033.1 | 398, 1-146  432, 124-267 | Complete FHb  Unknown N-terminal + incomplete FHb |
| *Candida parapsilopsis* | 13.1 C | [CPAG_00481](http://supfam.mrc-lmb.cam.ac.uk/SUPERFAMILY/nolink.html)  [CPAG_03668](http://supfam.mrc-lmb.cam.ac.uk/SUPERFAMILY/nolink.html) | 397, 9-155  419, 129-299 | Complete FHb  Unknown N-terminal + incomplete FHb |
| *Candida tropicalis* | 14.6 C | XP_002548361.1 XP_002548363.1 XP_002548362.1 XP_002546384.1  XP_002546377.1  XP_002550469.1  [CTRG_01406.3](http://www.broad.mit.edu/annotation/genome/candida_tropicalis/FeatureSearch.html?formids=If%2CHidden%2CIf_0%2CIf_1%2CIf_2%2CIf_3%2CIf_4%2CIf_5%2CIf_6%2CPropertySelection%2CIf_7%2CIf_8%2CIf_9%2CIf_10%2CTextField%2CIf_11%2CIf_12%2CIf_13%2CIf_14%2CIf_15%2CIf_16%2CIf_17%2CTextField_0%2CIf_18%2CIf_19%2CTextField_1%2CIf_20%2CIf_21%2CIf_22%2CTextField_2%2CIf_23%2CIf_24%2CIf_25%2CTextField_3%2CIf_26%2CIf_27%2CIf_28%2CTextField_4%2CIf_29%2CIf_30%2CTextField_5%2CTextField_6%2CSubmit&component=searchForm.%24Form&service=direct&session=T&reservedids=dbAlias%2Cdomain%2ClocalName%2CobjectId&submitmode=submit&submitname=&If=F&Hidden=*5352_153&If_0=T&If_1=T&If_2=F&If_3=F&If_4=F&If_5=T&If_6=T&If_7=F&If_8=F&If_9=F&If_10=T&If_11=F&If_12=F&If_13=F&If_14=T&If_15=T&If_16=T&If_17=T&If_18=T&If_19=T&If_20=T&If_21=T&If_22=T&If_23=T&If_24=T&If_25=T&If_26=T&If_27=T&If_28=T&If_29=T&If_30=T&PropertySelection=Gene&TextField_0=CT3.G.CT3&TextField_1=C.+tropicalis&TextField_2=&TextField_3=&TextField_4=&TextField_5=1&TextField_6=14601835&Submit=Submit&TextField=CTRG_01406.3) | 390, 11-154  390, 11-154  394, 13-156  394, 13-156  397, 13-156  522, 230-407  964, 1-150 | Complete FHb  Complete FHb  Complete FHb  Complete FHb  Complete FHb  Unknown N-terminal + incomplete FHb FD + unknown C-terminal |
| *Vanderwaltozyma polyspora*  *(*Kluyveromyces polysporus) | 14.7 C | XP_001644502.1  XP_00164706.1 | 394, 152-281  398, 1-148 | Unknown N-terminal + incomplete FHb Complete FHb |
| *Zygosaccharomyces rouxii* | 9.8 C | XP_002495591.1  XP_002497875.1 | 396, 1-147  455, 177-321 | Complete FHb  Unknown N-terminal + incomplete FHb |
| [**Taphrinomycotina**](javascript:void(0))  **Pneumocystidomycetes**  **Pneumocystidales**  **Pneumocystidaceae** |  |  |  |  |
| *Pneumocystis carinii* | 7.7 | No globins |  |  |
| *Pneumocystis jirovecii* | 7.5 | No globins |  |  |
| *Pneumocystis murina* | 6.5 | No globins |  |  |
| **Schizosaccharomycetes (Sch)**  **Schizosaccharomycetales** |  |  |  |  |
| *Schizosaccharomyces cryophilus* | ? | ACQJ01000207.1 |  | FHb |
| *Schizosaccharomyces octosporus* | 11.2 | SOCG 0365 | 423, 25-172 | Complete FHb |
| *Schizosaccharomyces pombe* | 12.5 C | NP_595017.1 | 427, 31-178 | FHb with N-terminal signal sequence |
| *Schizosaccharomyces sp.OY26* | ? | FHb |  |  |
| **Mitosporic Ascomycota** |  |  |  |  |
| *Thermomyces (Humicola) lanuginosa* | ? | TlanSEQ21961 |  | FHb |
| **Basidiomycota (BAS)**  **Agaricomycotina (Aga)**  **Agaricomycetes**  **Agaricomycetidae**  **Agaricales** (gill mushrooms) |  |  |  |  |
| **Agaricaceae** |  |  |  |  |
| *Agaricus bisporus* | 30.2 C | No globins? |  |  |
| **Marasmiaceae** |  |  |  |  |
| *Moniliophthora perniciosa* | 39 | No globins? |  |  |
| **Pleurotales** |  |  |  |  |
| *Pleurotus ostreatus* | 34.3 | No globins? |  |  |
| **Psathyrellaceae** |  |  |  |  |
| *Coprinopsis cinerea* | 36.3 C | XP_001838134.1  XP_001836632.1 ` | 235, 1-194  391, 113-278 | Sgb  SD + unknown C-terminal |
| **Schizophyllaceae** |  |  |  |  |
| *Schizophyllum commune* | 38.5 C | EFI96709.1  jgi\|Schco1\|43290\|  XP_003038598.1 | 416, 1-146  199, 25-199  254, 78-254 | Complete FHb  Sgb  Sgb |
| **Tricholomataceae** |  |  |  |  |
| *Laccaria bicolor* | 64.9 C | Gb\|ABFE01002552.1 |  | Sgb |
| **Boletales** |  |  |  |  |
| *Coniophora puteana* | 43.0 | jgi\|Conpu1\|93014 | 434, 14-160 | Complete FHb |
| *Serpula lacrymans* | 42.8 | No globins? |  |  |
| **Agaricomycetes incertae sedis** |  |  |  |  |
| **Corticiales** |  |  |  |  |
| *Phanerochaete chrysosporium* | 35.2 C | jgi\|Phchr1\|122095  PchrSEQ011386 | 421, 17-167 | Complete FHb  Sgb |
| *Punctularia strigosozonata* | 34.2 | jgi\|Punst1\|10076  jgi\|Punst1\|104235 | 433, 13-159  305, 120-305 | Complete FHb  Sgb |
| **Gloeophyllales** |  |  |  |  |
| *Gloeophyllum trabeum* | 37.2 | GtraSEQ10775 |  | Sgb |
| **Hymenochaetales** |  |  |  |  |
| *Formitiporia mediterranea* | 63.4 | No globins? |  |  |
| **Polyporales** |  |  |  |  |
| *Dichomitus squalens* | 42.8 | jgi\|Disq1\|148805  jgi\|Disq1\|145813 | 424, 16-153  251, 74-251 | Complete FHb  Sgb |
| *Fomitopsis pinicola* | 46.3 | jgi\|Fompi1\|62952  jgi\|Fompi1}160179 | 416, 2-151  263, 85-263 | Complete FHb  Sgb |
| *Postia placenta* | 90.9 C | ABWF01001276.1 |  | Sgb |
| *Trametes versicolor* | 44.8 | jgi\|Trave1\|136661  jgi\|Trave1\|68714  jgi\|Trave1\|136661 | 430, 11-157  433, 11-157  248, 71-248 | Complete FHb  Complete FHb  Sgb |
| *Wolfiporia cocos* | 50.5 | No globins? |  |  |
| **Russulales** |  |  |  |  |
| *Heterobasidion annosum* | 33.7 | jgi\|Hetan1\|145293 | 251, 34-213 | Sgb |
| *Stereum hirsutum* | 46.5 | jgi\|Stehi1\|160474  jgi\|Stehi1\|5037 | 392, 1-120  202, 27-202 | FHb missing helices A and B  Sgb |
| **Tremellomycetes**  **Tremellales** |  |  |  |  |
| **Tremellaceae**  *Filobasidiella neoformans*  *(Cryptococcus neoformans)* | 18.5 - 19.5 C | XP_569844.1 | 504, 80-212 | FHb with N-terminal signal sequence |
| *Cryptococcus bacillisporus (gattii)* | ~20 C | AAFP01000329.1 |  | FHb |
| *Tremella mesenterica* | 28.6 | No globins? |  |  |
| **Pucciniomycotina (Puc)** |  |  |  |  |
| **Microbotryomycetes** |  |  |  |  |
| *Microbotryum violaceum* | ? | No globins? |  |  |
| *Sporobolomyces roseus* | 21.2 | No globins? |  |  |
| *Rhodotorula graminis* | 21 | No globins? |  |  |
| **Mixiomycetes** |  |  |  |  |
| *Mixia osmundae* | ? | No globins? |  |  |
| **Pucciniomycetes**  **Pucciniales** (rusts) |  |  |  |  |
| *Melamspora laricis-populina* | 101.1 | No globins? |  |  |
| *Phakopsora pachyrhizi* | 50 | No globins? |  |  |
| *Puccinia graminis* | 88.6 | EFP84457.1 | 363, 28-212 | SD + unknown C-terminal |
| **Ustilaginomycotina (Ust)** |  |  |  |  |
| **Exobasidiomycetes**  **Malasseziales** |  |  |  |  |
| *Malassezia globosa* | 9.0 C | XP_001730006.1 | 409, 7-156 | Complete FHb |
| *Malassezia restricta* | 9.0 C | AAXK01002605.1 |  | FHb |
| **Ustilaginomycetes**  **Ustilaginales** |  |  |  |  |
| *Ustilago maydis (zeae)* | 19.7 C | No globins |  |  |
| **Basidiomycota incertae sedis** |  |  |  |  |
| *Wallemia sebi* | 9.82 | jgi\|Walsel30995  jgi\|Walse1\|8099  jgi\|Walse1\|13193 | 298, 8-138  383, 1-140  395, 6-151 | Incomplete FHb  Complete FHb  Complete FHb |
| **Glomeromycota**  **Glomeromycetes**  **Glomerales** |  |  |  |  |
| *Glomus intraradices* | 14.4 | No globins |  |  |
| **Fungi incertae sedis (FINS)**  **Basal fungal lineages** |  |  |  |  |
| **Mucoromycotina** |  |  |  |  |
| *Cunninghamella elegans* | ? | CeleSEQ13003 |  | Sgb |
| *Mucor circinelloides* | 36.6 | jgi\|Mucci1\|88439\|  jgi\|Mucci1\|77690\|  jgi\|Mucci1\|25566\|  jgi\|Mucci1\|42847 | 148  278, 103-243  240, 1-188  245, 1-188 | Fgb  Unknown N-terminal + FD  Sgb  Sgb |
| *Phycomyces blakesleeanus* | 55.85 | jgi\|Phybl1\|68682  jgi\|Phybl1\|78799  jgi\|Phybl1\|80048 | 408, 249-383  339, 182-320  228, 1-187 | Unknown N-terminal + FD  Unknown N-terminal + FD  Sgb |
| *Rhizopus oryzae* | 40 | [RO3G_14185.1](http://www.broad.mit.edu/annotation/genome/rhizopus_oryzae/FeatureSearch.html?formids=If%2CHidden%2CIf_0%2CIf_1%2CIf_2%2CIf_3%2CIf_4%2CIf_5%2CIf_6%2CPropertySelection%2CIf_7%2CIf_8%2CIf_9%2CIf_10%2CTextField%2CIf_11%2CIf_12%2CIf_13%2CIf_14%2CIf_15%2CIf_16%2CIf_17%2CTextField_0%2CIf_18%2CIf_19%2CTextField_1%2CIf_20%2CIf_21%2CIf_22%2CTextField_2%2CIf_23%2CIf_24%2CIf_25%2CTextField_3%2CIf_26%2CIf_27%2CIf_28%2CTextField_4%2CIf_29%2CIf_30%2CTextField_5%2CTextField_6%2CSubmit&component=searchForm.%24Form&service=direct&session=T&reservedids=dbAlias%2Cdomain%2ClocalName%2CobjectId&submitmode=submit&submitname=&If=F&Hidden=*5354_101&If_0=T&If_1=T&If_2=F&If_3=F&If_4=F&If_5=T&If_6=T&If_7=F&If_8=F&If_9=F&If_10=T&If_11=F&If_12=F&If_13=F&If_14=T&If_15=T&If_16=T&If_17=T&If_18=T&If_19=T&If_20=T&If_21=T&If_22=T&If_23=T&If_24=T&If_25=T&If_26=T&If_27=T&If_28=T&If_29=T&If_30=T&PropertySelection=Gene&TextField_0=RO3.G.RO3&TextField_1=R.+oryzae&TextField_2=&TextField_3=&TextField_4=&TextField_5=1&TextField_6=49912738&Submit=Submit&TextField=RO3G_14185.1)  [RO3G_01994.1](http://www.broad.mit.edu/annotation/genome/rhizopus_oryzae/FeatureSearch.html?formids=If%2CHidden%2CIf_0%2CIf_1%2CIf_2%2CIf_3%2CIf_4%2CIf_5%2CIf_6%2CPropertySelection%2CIf_7%2CIf_8%2CIf_9%2CIf_10%2CTextField%2CIf_11%2CIf_12%2CIf_13%2CIf_14%2CIf_15%2CIf_16%2CIf_17%2CTextField_0%2CIf_18%2CIf_19%2CTextField_1%2CIf_20%2CIf_21%2CIf_22%2CTextField_2%2CIf_23%2CIf_24%2CIf_25%2CTextField_3%2CIf_26%2CIf_27%2CIf_28%2CTextField_4%2CIf_29%2CIf_30%2CTextField_5%2CTextField_6%2CSubmit&component=searchForm.%24Form&service=direct&session=T&reservedids=dbAlias%2Cdomain%2ClocalName%2CobjectId&submitmode=submit&submitname=&If=F&Hidden=*5354_101&If_0=T&If_1=T&If_2=F&If_3=F&If_4=F&If_5=T&If_6=T&If_7=F&If_8=F&If_9=F&If_10=T&If_11=F&If_12=F&If_13=F&If_14=T&If_15=T&If_16=T&If_17=T&If_18=T&If_19=T&If_20=T&If_21=T&If_22=T&If_23=T&If_24=T&If_25=T&If_26=T&If_27=T&If_28=T&If_29=T&If_30=T&PropertySelection=Gene&TextField_0=RO3.G.RO3&TextField_1=R.+oryzae&TextField_2=&TextField_3=&TextField_4=&TextField_5=1&TextField_6=49912738&Submit=Submit&TextField=RO3G_01994.1)  [RO3G_09859.1](http://www.broad.mit.edu/annotation/genome/rhizopus_oryzae/FeatureSearch.html?formids=If%2CHidden%2CIf_0%2CIf_1%2CIf_2%2CIf_3%2CIf_4%2CIf_5%2CIf_6%2CPropertySelection%2CIf_7%2CIf_8%2CIf_9%2CIf_10%2CTextField%2CIf_11%2CIf_12%2CIf_13%2CIf_14%2CIf_15%2CIf_16%2CIf_17%2CTextField_0%2CIf_18%2CIf_19%2CTextField_1%2CIf_20%2CIf_21%2CIf_22%2CTextField_2%2CIf_23%2CIf_24%2CIf_25%2CTextField_3%2CIf_26%2CIf_27%2CIf_28%2CTextField_4%2CIf_29%2CIf_30%2CTextField_5%2CTextField_6%2CSubmit&component=searchForm.%24Form&service=direct&session=T&reservedids=dbAlias%2Cdomain%2ClocalName%2CobjectId&submitmode=submit&submitname=&If=F&Hidden=*5354_101&If_0=T&If_1=T&If_2=F&If_3=F&If_4=F&If_5=T&If_6=T&If_7=F&If_8=F&If_9=F&If_10=T&If_11=F&If_12=F&If_13=F&If_14=T&If_15=T&If_16=T&If_17=T&If_18=T&If_19=T&If_20=T&If_21=T&If_22=T&If_23=T&If_24=T&If_25=T&If_26=T&If_27=T&If_28=T&If_29=T&If_30=T&PropertySelection=Gene&TextField_0=RO3.G.RO3&TextField_1=R.+oryzae&TextField_2=&TextField_3=&TextField_4=&TextField_5=1&TextField_6=49912738&Submit=Submit&TextField=RO3G_09859.1) | 236, 36-213  233, 47-210  238, 13-187 | Fgb  Fgb  Sgb |
| **Neocallimastigomycota** |  | ? |  |  |
| **Microsporidia** |  |  |  |  |
| **Apansporoblastina** |  |  |  |  |
| **Enterocytozoonidae**  *Enterocytozoon bieneusi* | 6.0 | No globins |  |  |
| **Nosematidae**  *Nosema ceranae* | 7-15 | No globins |  |  |
| **Unikaryonidae**  *Encephalitozoon cuniculi*  *Encephalitozoon intestinalis* | 2.9 C  2.0 C | No globins  No globins |  |  |
| **Microsporidia incertae sedis**  *Antonospora locustae*  *Nematocida parisii*  *Octosporea bayeri* | 2.0  ?  24 | No globins  No globins  No globins |  |  |

^1^ Based on SUPERFAMILY globin gene assignments (supfam.cs.bris.ac.uk/SUPERFAMILY) and sequences found via BLASTP searches of the Fungal Genomes Central at NCBI (<http://www.ncbi.nlm.nih.gov/projects/genome/guide/fungi>) and Washington University Genome Institute (genome.wustl.edu/genomes/fungi). Globins missing an entry in column 4, were identified via TBLASTN searches at the NCBI site (http://www.ncbi.nlm.nih.gov/sutils/genom_table.cgi?organism=fungi).

^2^ Based on the NCBI taxonomy site (<http://www.ncbi.nlm.nih.gov/Taxonomy/>); number of subspecies in parentheses. The first three letters of the underlined taxon names are used in the identification of globin sequences

^3^ From Kullman B, Tamm H, Kullman K: Fungal Genome Size Database, 200*5* (<http://www.zbi.ee/fungal-genomesize>) and the JGI Fungi Portal (http:// genome.jgi-psf.org/programs/fungi/index.jsf).

^4^ FHb – flavohemoglobin, chimeric proteins (~400aa) comprising a 3/3 N-terminal globin and a flavin reductase domain; FD – FHb-like globin domain; Fgb – single domain FHb-like globin; SD – sensor globin domain; Sgb – single domain sensor globin, <260aa; Tgb – truncated 2/2 myoglobin fold globin family, comprises groups 1, 2 and 3. Where the N- and C-terminal extensions are >100aa, they are considered to represent potential protein domains.
