# Supplementary material for: A Phylogenetic Analysis of the Globins in Fungi
Source: PLoS One. 2012 Feb 27;7(2):e31856. doi: 10.1371/journal.pone.0031856 (PMC3287990; doi:10.1371/journal.pone.0031856)
Supplement: Table S2 — MUMSA scores for the five multiple alignment algorithms used in this work. (DOCX) [file pone.0031856.s012.docx]

Table S2. MUMSA scores for the five multiple alignment algorithms used in this work.

| **Analysis** | **Alignment algorithm** | **MUMSA score** |
| --- | --- | --- |
| fungal FHbs *(Figure 3)* | MAFFT | 0.938381 |
|  | T-coffee | 0.913813 |
|  | PROBCONS | 0.910271 |
|  | MUSCLE | 0.891018 |
|  | COBALT | 0.878450 |
| fungal and bacterial FHbs *(Figure 4)* | T-coffee | 0.951429 |
|  | PROBCONS | 0.938035 |
|  | MAFFT | 0.938922 |
|  | MUSCLE | 0.929305 |
|  | COBALT | 0.906212 |
| fungal Sgbs *(Figure 5)* | T-coffee | 0.956388 |
|  | MUSCLE | 0.943880 |
|  | PROBCONS | 0.940100 |
|  | MAFFT | 0.932803 |
|  | COBALT | 0.923393 |
| bacterial GCSs and Sgbs *(Suppl Figure7)* | T-coffee | 0.921816 |
|  | MUSCLE | 0.906534 |
|  | MAFFT | 0.901961 |
|  | PROBCONS | 0.897240 |
|  | COBALT | 0.867614 |
| fungal and bacterial Sgbs *(Figure 6)* | T-coffee | 0.894987 |
|  | PROBCONS | 0.861631 |
|  | MUSCLE | 0.861279 |
|  | MAFFT | 0.851032 |
|  | COBALT | 0.843564 |
| fungal and bacterial T1 globins *(Figure 7)* | T-coffee | 0.934989 |
|  | PROBCONS | 0.923199 |
|  | MUSCLE | 0.920765 |
|  | MAFFT | 0.913204 |
|  | COBALT | 0.896256 |
