# Supplementary material for: A Phylogenetic Analysis of the Globins in Fungi
Source: PLoS One. 2012 Feb 27;7(2):e31856. doi: 10.1371/journal.pone.0031856 (PMC3287990; doi:10.1371/journal.pone.0031856)
Supplement: Table S3 — Hits obtained via PSIBLAST 2nd iteration using Aspergillus clavatus (Ascomycota) FHb globin domain (XP_001274889.1), as query, and selecting the first 40 fungal FHbs for the 2nd iteration. (DOCX) [file pone.0031856.s013.docx]

Table S2. Hits obtained via PSIBLAST 2 ^nd^ iteration using *Aspergillus clavatus* (Ascomycota) FHb globin domain (XP_001274889.1), as query, and selecting the first 40 fungal FHbs for the 2^nd^ iteration.

| Name | Taxon | Identification | Bit score | E-value |
| --- | --- | --- | --- | --- |
| 53 Fungal FHbs |  |  |  |  |
| *Burkholderia sp. CCGE1003* | Betaproteobacteria, Burkholderiales | XP_003905744 | 216 | 8e-55 |
| *Burkholderia graminis* | Betaproteobacteria, Burkholderiales | EDT11733 | 215 | 1e-54 |
| 3 Fungal FHbs | Betaproteobacteria, Burkholderiales |  |  |  |
| *Burkholderia oklahomensis* | Betaproteobacteria, Burkholderiales | ZP_02356913 | 208 | 2e-52 |
| *Deinococcus radiodurans* | Deinococcus | NP_285566 | 206 | 8e-52 |
| *Burkholderia ambifaria* | Betaproteobacteria, Burkholderiales | ZP_02908856 | 205 | 1e-51 |
| 23 Burkholderiales | Betaproteobacteria, Burkholderiales |  |  |  |
| 1 Fungal FHb |  |  |  |  |
| *Methylotenera sp. 301* | Betaproteobacteria, Methylophilales |  | 199 | 2e-49 |
| *Bacillus sp. B14905* | Firmicutes, Bacillales | EAZ86663 | 198 | 2e-49 |
| *Lysinibacillus sphaericus* | Firmicutes, Bacillales | ACA41869 | 197 | 4e-49 |
| *Sinorhizobium meliloti* | Alphaproteobacteria, Rhizobiales | ZP_07584926 | 196 | 6e-49 |
| 47 Fungal FHbs and >600 bacterial FHbs and Sgbs, including eukaryote 3/3 Hbs | | | | |
| *Gallus gallus* Ngb | Vertebrata, Aves | ABS87379 | 56.1 | 1e-06 |
| >500 bacterial, metazoan, | plant and other eukaryote 3/3 globins |  |  |  |
