# Supplementary material for: A Phylogenetic Analysis of the Globins in Fungi
Source: PLoS One. 2012 Feb 27;7(2):e31856. doi: 10.1371/journal.pone.0031856 (PMC3287990; doi:10.1371/journal.pone.0031856)
Supplement: Table S4 — Hits obtained via PSIBLAST 2nd iteration using Ajellomyces dermatidis (Ascomycota; Pezizomycotina; Eurotiomycetes) Sgb, 214aa (33–214) (XP_002625170.1), as query. (DOCX) [file pone.0031856.s014.docx]

TableS3. Hits obtained via PSIBLAST 2^nd^ iteration using *Ajellomyces dermatidis* ([Ascomycota](javascript:void(0)); [Pezizomycotina](javascript:void(0)); [Eurotiomycetes](javascript:void(0))) Sgb, 214aa (33-214) (XP_002625170.1), as query.

| Name | Taxon | Identification | Bit score | E-value |
| --- | --- | --- | --- | --- |
| 57 Fungal Sgbs |  |  |  |  |
| *Naegleria gruberi* | Heterolobosa | XP_002678116 | 189 | 1e-46 |
| *Pirellula staleyi* | Planctomycete | ADB17426 | 168 | 3e-40 |
| *Haliangium ochraceum* | Deltaproteobacteria | ACY17660 | 162 | 2e-38 |
| *Philodina roseola* | Rotifera | ACD54784 | 160 | 1e-37 |
| *Planctomyces maris* | Planctomycete | EDL57731 | 154 | 8e-36 |
| *Blastopirellula marina* | Planctomycete | EAQ81802 | 137 | 1e-31 |
| *Candidatus Kuenenia stuttgartiensis* | Planctomycete | CAJ72898 | 50.7 | 1e-04 |
| *Desulfotalea psychrophila* | Deltaproteobacteria | CAG34930 | 46.1 | 0.003 |
