# Supplementary material for: A Phylogenetic Analysis of the Globins in Fungi
Source: PLoS One. 2012 Feb 27;7(2):e31856. doi: 10.1371/journal.pone.0031856 (PMC3287990; doi:10.1371/journal.pone.0031856)
Supplement: Table S5 — Hits obtained via PSIBLAST 2nd iteration using Coprinopsis cinerea (Basidiomycota) Sgb, 235aa (26–194) (XP_001838134.1), as query. (DOCX) [file pone.0031856.s015.docx]

Table S5. Hits obtained via PSIBLAST 2nd iteration using *Coprinopsis cinerea* (Basidiomycota) Sgb, 235aa (26-194) (XP_001838134.1), as query.

| Name | Taxon | Identification | Bit score | E-value |
| --- | --- | --- | --- | --- |
| 58 Fungal Sgbs |  |  |  |  |
| *Naegleria gruberi* | Heterolobosa | XP_002678116 | 209 | 2e-52 |
| *Pirellula staleyi* | Planctomycete | ADB17426 | 179 | 3e-43 |
| *Haliangium ochraceum* | Deltaproteobacteria | ACY17660 | 175 | 4e-42 |
| 1 Fungal Sgb |  |  |  |  |
| *Planctomyces maris* | Planctomycete | EDL57731 | 170 | 9e-41 |
| *Philodina roseola* | Rotifera | ACD54784 | 162 | 3e-38 |
| *Blastopirellula marina* | Planctomycete | EAQ81802 | 152 | 3e-35 |
| 2 Fungal Sgbs |  |  |  |  |
| *Candidatus Kuenenia stuttgartiensis* | Planctomycete | CAJ72898 | 54.5 | 9e-06 |
| *Desulfotalea psychrophila* | Deltaproteobacteria | CAG34930 | 49.9 | 3e-04 |
