# Supplementary material for: A Phylogenetic Analysis of the Globins in Fungi
Source: PLoS One. 2012 Feb 27;7(2):e31856. doi: 10.1371/journal.pone.0031856 (PMC3287990; doi:10.1371/journal.pone.0031856)
Supplement: Table S6 — Hits obtained via BLASTP using the T1 globin domain from the Allomyces macrogynus (Blastocladiomycota) 1129aa chimeric protein (AMAG_16521T0), as query. (DOCX) [file pone.0031856.s016.docx]

TableS5. Hits obtained via BLASTP using the T1 globin domain from the *Allomyces macrogynus* (Blastocladiomycota) 1129aa chimeric protein (AMAG_16521T0), as query.

| Name | Taxon | Identification | Bit score | E-value |
| --- | --- | --- | --- | --- |
| *Tetrahymena thermophila*, 1156aa  globin domains 6, 2, 3, 5, 7, 8, 4 | Alveolata, Ciliophora | XP_001030649 | 83.2-43.5 | 1e-14-0.008 |
| 3 *Perkinsus marinus*, 118aa | Alveolata, Ciliophora | XP_002779628  XP_002779633  XP_002779040 | 77.0-75.1 | 7e-13-2e-12 |
| *Mycobacterium vanbaalenii*, 124aa | Actinobacteria, Actinomycetales | YP_953792 | 73.2 | 9e-12 |
| *Bacillus tusciae*, 129aa | Firmicutes, Bacillales | YP_003589034 | 72.4 | 2e-11 |
| *Halogeometricum borinquense*, 125aa | Euryarchaeota, Halobacteria | YP_004037121 | 71.2 | 4e-11 |
| *Cyanothece sp. PCC 7424*, 124aa | Cyanobacteria; Chroococcales | YP_002380387 | 69.3 | 1e-10 |
| *Perkinsus marinus*, 118aa | Alveolata | XP_002782190 | 68.9 | 2e-10 |
| *Arthrospira platensis*, 124aa | Cyanobacteria; Oscillatoriales | ZP_06382595 | 68.2 | 3e-10 |
| *Mycobacterium gilvum*, 124aa | Actinobacteria, Actinomycetales | YP_001134529 | 68.2 | 3e-10 |
