# Supplementary material for: A Phylogenetic Analysis of the Globins in Fungi
Source: PLoS One. 2012 Feb 27;7(2):e31856. doi: 10.1371/journal.pone.0031856 (PMC3287990; doi:10.1371/journal.pone.0031856)
Supplement: Table S7 — Hits obtained via BLASTP using the Batrachochytrium dendrobatidis (Chytridiomycota) T1 globin (BDEG_06358), as query. (DOCX) [file pone.0031856.s017.docx]

# Table S6. Hits obtained via BLASTP using the *Batrachochytrium dendrobatidis* (Chytridiomycota) T1 globin ([BDEG_06358](http://www.broad.mit.edu/annotation/genome/batrachochytrium_dendrobatidis/FeatureSearch.html?formids=If%2CHidden%2CIf_0%2CIf_1%2CIf_2%2CIf_3%2CIf_4%2CIf_5%2CIf_6%2CPropertySelection%2CIf_7%2CIf_8%2CIf_9%2CIf_10%2CTextField%2CIf_11%2CIf_12%2CIf_13%2CIf_14%2CIf_15%2CIf_16%2CIf_17%2CTextField_0%2CIf_18%2CIf_19%2CTextField_1%2CIf_20%2CIf_21%2CIf_22%2CTextField_2%2CIf_23%2CIf_24%2CIf_25%2CTextField_3%2CIf_26%2CIf_27%2CIf_28%2CTextField_4%2CIf_29%2CIf_30%2CTextField_5%2CTextField_6%2CSubmit&component=searchForm.%24Form&service=direct&reservedids=dbAlias%2Cdomain%2ClocalName%2CobjectId&submitmode=submit&submitname=&If=F&Hidden=*5348_161&If_0=T&If_1=T&If_2=F&If_3=F&If_4=F&If_5=T&If_6=T&If_7=F&If_8=F&If_9=F&If_10=T&If_11=F&If_12=F&If_13=F&If_14=T&If_15=T&If_16=T&If_17=T&If_18=T&If_19=T&If_20=T&If_21=T&If_22=T&If_23=T&If_24=T&If_25=T&If_26=T&If_27=T&If_28=T&If_29=T&If_30=T&PropertySelection=Gene&TextField_0=BD_JEL423.G.BD_JEL423&TextField_1=B.+dendrobatidis&TextField_2=&TextField_3=&TextField_4=&TextField_5=1&TextField_6=23790384&Submit=Submit&TextField=BDEG_06358)), as query.

| Name | Taxon | Identification | Bit score | E-value |
| --- | --- | --- | --- | --- |
| *Bacillus tusciae*, 129aa | Firmicutes, Bacillales | YP_003589034 | 106 | 8e-22 |
| *Brevibacillus brevis*, 118aa | Firmicutes, Bacillales | YP_002774071 | 98.2 | 3e-19 |
| *Bacillus megaterium*. 120aa | Firmicutes, Bacillales | YP_003562873 | 97.8 | 4e-19 |
| *Microcoleus chthonoplastes*, 123aa | Cyanobacteria; Oscillatoriales | ZP_05025218 | 97.8 | 4e-19 |
| *Coraliomargarita akajimensis*, 123aa | Verrucomicrobia; Opitutae | YP_003548557 | 97.8 | 4e-19 |
| *Methylomonas sp. 16a*, 125aa | Gammaproteobacteria; Methylococcales | ABI17848 | 97.4 | 4e-19 |
| 3 *Tetrahymena pyriformis*, 121aa | Alveolata; Ciliophora | P17724  XP_001023424  Q03459 | 96.7-96.3 | 8e-19-1e-18 |
